# CD69 Is a TGF-β/1α,25-dihydroxyvitamin D_3_ Target Gene in Monocytes

**DOI:** 10.1371/journal.pone.0064635

**Published:** 2013-05-16

**Authors:** Thea K. Wöbke, Andreas von Knethen, Dieter Steinhilber, Bernd L. Sorg

**Affiliations:** 1 Institute of Pharmaceutical Chemistry/ZAFES (Zentrum für Arzneimittelforschung, Entwicklung und Sicherheit), Goethe-University, Frankfurt am Main, Germany; 2 Institute of Biochemistry I-Pathobiochemistry, Faculty of Medicine, Goethe-University, Frankfurt am Main, Germany; Virgen Macarena University Hospital, Spain

## Abstract

CD69 is a transmembrane lectin that can be expressed on most hematopoietic cells. In monocytes, it has been functionally linked to the 5-lipoxygenase pathway in which the leukotrienes, a class of highly potent inflammatory mediators, are produced. However, regarding CD69 gene expression and its regulatory mechanisms in monocytes, only scarce data are available. Here, we report that CD69 mRNA expression, analogous to that of 5-lipoxygenase, is induced by the physiologic stimuli transforming growth factor-β (TGF-β) and 1α,25-dihydroxyvitamin D_3_ (1α,25(OH)_2_D_3_) in monocytic cells. Comparison with T- and B-cell lines showed that the effect was specific for monocytes. CD69 expression levels were increased in a concentration-dependent manner, and kinetic analysis revealed a rapid onset of mRNA expression, indicating that CD69 is a primary TGF-β/1α,25(OH)_2_D_3_ target gene. PCR analysis of different regions of the CD69 mRNA revealed that *de novo* transcription was initiated and proximal and distal parts were induced concomitantly. In common with 5-lipoxygenase, no activation of 0.7 kb or ∼2.3 kb promoter fragments by TGF-β and 1α,25(OH)_2_D_3_ could be observed in transient reporter assays for CD69. Analysis of mRNA stability using a transcription inhibitor and a 3′UTR reporter construct showed that TGF-β and 1α,25(OH)_2_D_3_ do not influence CD69 mRNA stability. Functional knockdown of Smad3 clearly demonstrated that upregulation of CD69 mRNA, in contrast to 5-LO, depends on Smad3. Comparative studies with different inhibitors for mitogen activated protein kinases (MAPKs) revealed that MAPK signalling is involved in CD69 gene regulation, whereas 5-lipoxygenase gene expression was only partly affected. Mechanistically, we found evidence that CD69 gene upregulation depends on TAK1-mediated p38 activation. In summary, our data indicate that CD69 gene expression, conforming with 5-lipoxygenase, is regulated monocyte-specifically by the physiologic stimuli TGF-β and 1α,25(OH)_2_D_3_ on mRNA level, although different mechanisms account for the upregulation of each gene.

## Introduction

The transmembrane lectin CD69 is best characterized and widely used as an early T-lymphocyte activation marker that is expressed upon inflammatory stimuli [1]. A major function of CD69 is to shut down lymphocyte egress from lymphoid organs via inhibition of sphingosine 1-phosphate signalling [2]. However, CD69 expression has not only been found on lymphocytes, but on all bone marrow-derived cells except erythrocytes (reviewed in [1]). Regarding its expression on monocytic cells, one report exists that describes constitutive expression on CD14 positive monocytes [3], but in a subsequent study only 10% of total monocytes were found to be positive for CD69. In that study the basal level of CD69 was enhanced by stimulation with leptin, lipopolysaccharide or phorbol 12-myristate 13-acetate (PMA) [4].

With respect to its role in monocytes, CD69 has been functionally linked to 5-lipoxygenase (5-LO), the key enzyme in the conversion of arachidonic acid to leukotrienes [3]. Leukotrienes are potent lipid mediators involved in inflammatory disorders, including asthma, arthritis as well as allergic reactions, and have been implicated in the pathogenesis of atherosclerosis and different neoplasms [5]. Cross-linking of CD69 on monocytes coincided with Ca^2+^ influx, arachidonic acid release and leukotriene B_4_ production [3]. Moreover, induction of apoptosis by anti-CD69 antibodies in LPS-stimulated human monocytes or monocytic THP-1 could be blocked by 5-LO inhibitors [6]. 5-lipoxygenase is a known TGF-β/1α,25(OH)_2_D_3_ target gene in monocytes [7], [8], and several other genes are established to be regulated by this combination of chemically unrelated mediators [9]. The signalling pathways of TGF-β and 1α,25(OH)_2_D_3_ alone are well understood, respectively. The lipophilic hormone 1α,25(OH)_2_D_3_ acts on mRNA expression via its nuclear receptor, the vitamin D receptor (VDR). Together with its heterodimeric binding partner, the retinoid X receptor (RXR), VDR binds to vitamin D responsive elements (VDREs) in regulatory DNA regions. Upon ligand binding, a complex of coactivator proteins is recruited, which subsequently acts on the basal transcription machinery [10]. On the other hand, the cell-impermeant peptide TGF-β signals through a specific cell surface receptor, the TGF-β receptor complex. Activation regulates mRNA biosynthesis either via the canonical Smad transcription factor pathway [11], or via non-Smad signalling pathways in which TGF-β activated kinase 1 (TAK1) is a central component and other mitogen activated protein kinases (MAPKs) like p38, Jnk and Erk are major players [11], [12], [13]. Smad proteins bind to their cognate binding elements on the DNA in cooperation with other transcription factors, where the complexes interact with the basal transcription machinery, whereas the signals of the non-Smad pathways are channelled through specific transcription factors that receive the signals of the different kinases. TGF-β and 1α,25(OH)_2_D_3_ share common effects on cell growth and differentiation, but the exact mechanisms of their interaction still remain to be elucidated.

Regarding mechanisms of CD69 gene regulation in monocytes, no systematic studies are available to date. We report here that CD69 is a novel TGF-β/1α,25(OH)_2_D_3_ target gene that is cell type specifically regulated in monocytic cells, but exhibits differences in the profile and the underlying mechanisms of gene regulation as compared to 5-LO. The main distinctions are that CD69 gene regulation by TGFβ/1α,25(OH)_2_D_3_, in contrast to 5-LO, depends on Smad3 and seems to be regulated by a signalling axis involving TAK1-mediated p38 activation.

## Materials and Methods

### Plasmid constructs

The following plasmids were generated by PCR amplification of the corresponding genomic regions from cDNA or genomic DNA (both generated from THP-1 cells), double digest and subsequent insertion into the respective parental vectors using standard cloning procedures: pcDNA-CD69, pcDNA-CD69 Intron 1, pcDNA-CD69 Exon 1–Intron 1, pcDNA-B2M, pGL3-CD69 0.650 and pGL3-CD69 2.26. In order to obtain plasmid pGL3-Prom CD69 3UTR, the 3′ end of the luciferase coding sequence was removed from the parental vector by double digest and reinserted as a PCR fragment, including an additional restriction site, together with the CD69 3′UTR PCR fragment. The PCR primer sequences, parental vectors and restriction sites used for insertion are summarized in Table 1. Plasmid pcDNA3.1-5LO [14] was a kind gift of Dr. O. Rådmark (Stockholm, Sweden). Reporter constructs p3TP-Lux [15], pGL3-basic-(CAGA)12MLP-luc [16], p(DR3)_4_ tk luc [17] were kindly provided by Dr. J. Massagué (New York, USA), Dr. C.-H. Heldin (Uppsala, Sweden) and Dr. C. Carlberg (Luxembourg), respectively. Vectors pSG5 VDR and pSG5 RXR [18], were a kind gift of Dr. C. Carlberg (Luxembourg), plasmids pCGN-Smad3 and pCGN-Smad4 [19] were kindly provided by Dr. X. F. Wang (Durham, USA).

**Table 1:** Oligonucleotides used for cloning by PCR.

Gene	Sequence of oligonucleotides used for cloninga)	Parental vector	Restriction sites	Name of target vector
CD69	F: ATAGCTAGCACCATGAGCTCTGAAAATTGTTTCGTAGC R: TTGAATTC CCTTATTATTTGTAAGGTTTGTTACATATCCAG	pcDNA 3.1 (+) (Invitrogen)	NheI/EcoRI	pcDNA CD69
B2M	F: CACGCTAGCTGCTGTCTCCATGTTTGATGTATCT R: GACGAATTCTCTCTGCTCCCCACCTCTAAGT	pcDNA 3.1 (+) (Invitrogen)	NheI/EcoRI	pcDNA B2M
CD69	F: CTTGCTAGCCAGAGAACAGCTCTTTGCATCCG R: CTCGAATTCCTCACTTCCTTCCCTAACAACCATTAG	pcDNA 3.1 (+) (Invitrogen)	NheI/EcoRI	pcDNA Exon 1-Intron 1
CD69	F: GTTGCTAGCCTGGAACCCACGGGTCACTGG R: GACGAATTCTGGAAGCCGCCTTTGCTACC	pcDNA 3.1 (+) (Invitrogen)	NheI/EcoRI	pcDNA Intron 1
CD69	F: TGCTAGCGCATAGCAAGGAAGTTCCAGACCAC R: GTCCATGG CTTTATTCTCAAGATTCCCTAGTTAATCTCAGG	pGL3-Basic (Promega)	NheI/NcoI	pGL3-CD69 0.650
CD69	F: TGCTAGCCGTGTACCAAACTGTCACTCCCTTCC R: GTCCATGG CTTTATTCTCAAGATTCCCTAGTTAATCTCAGG	pGL3-Basic (Promega)	NheI/NcoI	pGL3-CD69 2.26
Luciferase; CD69	F: CGTTCGTCACATCTCATCTACCTC R: CTGCTGCAG ATTACACGGCGATCTTTCCGCC; F: GACCTGCAGTAAGGAAACATGTTCACTTATTGACTATTATAG R: GAGTCTAGA GTGTTTATTCTACTTTTATTTCACATATATAAAAAC	pGL3-Promoter (Promega)	SphI/XbaI in the vector and PstI between the inserts	pGL3-Prom CD69 3UTR

a) F =  forward, R =  reverse; non-genomic sequences (overhangs, restriction sites, Kozak sequence) are underline.

### Cells and cell culture

The cell lines used in the study were obtained from DSMZ (Deutsche Sammlung von Mikroorganismen und Zellkulturen, Braunschweig, Germany). Mono Mac 6 cells (human acute monocytic leukemia) were maintained in RPMI 1640 medium supplemented with 10% (v/v) fetal calf serum, streptomycin (100 µg/ml), penicillin (100 U/ml), 1× non-essential amino acids, oxaloacetic acid (1 mM) and insulin (10 µg/ml). THP-1 (human acute monocytic leukemia), Jurkat (human T-cell leukemia), MOLT-4 (human T-cell leukemia) and Rec-1 (human B cell lymphoma) were maintained in RPMI 1640 medium supplemented with 10% (v/v) fetal calf serum, streptomycin (100 µg/ml) and penicillin (100 U/ml). HeLa (human cervix carcinoma) and 293T (human embryonic kidney) cells were cultured in Dulbecco's modified Eagle's medium supplemented with 10% (v/v) fetal calf serum, streptomycin (100 µg/ml), penicillin (100 U/ml) and sodium pyruvate (1 mM). The cells were grown in 100% humidity at 37°C, 5% CO_2_ for routine culture and at 6% CO_2_ for experimental use. For all experiments except luciferase assays, cells were cultured in medium containing charcoal-stripped FCS for 24 h prior to specific treatment.

### Real-time PCR (RT-qPCR)

Total RNA was extracted from the cells using TRIzol reagent® (Life Technologies) following the manufacturer's instructions, with some modifications: Cell pellets were lysed with TRIzol reagent using a 1 ml syringe with a 20 G Sterican hypodermic needle (Braun, Germany) and incubated at room temperature for 10 minutes. Then, 200 µl chloroform were added, samples were vortexed, incubated at room temperature for 15 minutes and centrifuged at 15000 g, 45 min, at 4°C. Clear supernatant (450 µl) was transferred into a new tube and 5 µl 3 M sodium acetate pH 6.5 and 500 µl isopropanol were added. After vortexing, RNA was allowed to precipitate for 3 h at –20°C. Samples were centrifuged 1 h at 15000 g, 4°C. RNA pellets were washed with 1 ml 75% ethanol and centrifuged 1 h at 15000 g, 4°C. Supernatants were removed and pellets were allowed to dry for a few minutes and dissolved in 50 µl DEPC-treated water (55°C, 10 min). RNA was quantified spectrophotometrically at 260 nm using a NanoPhotometer® (Implen, Munich, Germany). RNA integrity was verified by agarose gel electrophoresis. Subsequently, RNA was treated with DNaseI (Fermentas) to eliminate genomic DNA contamination. For cDNA synthesis, a constant amount of total RNA (2 µg) was reversely transcribed using the High Capacity RNA-to-cDNA Kit (Applied Biosystems) following the manufacturer's instructions. Absolute quantities (expressed as mRNA copies of the target gene per 10^6^ mRNA copies of the reference gene) were calculated from standard curves constructed from serial dilutions of linearized plasmids, which contained 100 ng/µl yeast tRNA as carrier. Data for standards and corresponding samples were always generated in the same PCR run. Standard plasmids were: pcDNA-CD69 (encoding full-length human CD69 cDNA), pcDNA Exon 1-Intron 1 and pcDNA Intron 1 (containing parts of the CD69 gene) pcDNA-B2M (containing parts of the human B2M coding sequence), pcDNA3.1-5LO (encoding full-length human 5-lipoxygenase), pCGN-Smad3 (encoding full-length human Smad3). B2M was chosen as reference gene after it had been determined as the most stable reference gene out of 5 candidates (β-actin, B2M, UBC, GAPDH, HPRT1) using geNorm software on datasets generated from THP1 and Mono Mac 6 cells that were treated with or without TGFβ/1α,25(OH)_2_D_3_. B2M mRNA levels remained unchanged upon TGFβ/1α,25(OH)_2_D_3_ or PMA treatment (data not shown). Primer pairs used for qPCR are listed in Table 2.

**Table 2:** Oligonucleotides used for RT-qPCR.

Gene	Sequence of oligonucleotide used for RT-qPCRa)
CD69 (Ex1/2-2)	F: GTGGACAAGAAAATGATGCC R: CATTCATTACAGCACACAG
CD69 (Ex4/5-Ex5/3′UTR)	F: ACAACTGGTTCAACGTTACAGG R: AGCAGCATCCACTGACACAG
CD69 (Intron 1)	F: CTGGAACCCACGGGTCACTGG R: TGGAAGCCGCCTTTGCTACC
CD69 (Exon 1- Intron 1)	F: CAGAGAACAGCTCTTTGCATCCG R: CTCACTTCCTTCCCTAACAACCATTAG
B2M	F: TGCTGTCTCCATGTTTGATGTATCT R: TCTCTGCTCCCCACCTCTAAGT
5-LO	F: GAATTACTCCAAAGCGATGG R: ATGACCCGCTCAGAAATAGTG
Smad3	F: GGCTGGAAGAAGGGCGAGCAG R: CAGGGACCTGGGGATGGTGATGC

a) F =  forward, R =  reverse.

### Luciferase assays

For luciferase assays, HeLa cells were transfected with reporter plasmids pGL3-CD69 0.650 (containing a 731 bp CD69 promoter fragment in front of the luciferase gene), pGL3-CD69 2.26 (2340 bp CD69 promoter fragment 5′ of luciferase), pGL3-Prom CD69 3UTR (CD69 3′UTR after the luciferase gene), p3TP-Lux (promoter with adjacent SBEs and TREs 5′ of luciferase), pGL3-basic-(CAGA)_12_MLP-luc (promoter with 12 concatemerized SBEs 5′ of luciferase), p(DR3)_4_ tk (promoter with four concatemerized VDREs 5′of luciferase) and pGL3-Basic (Promega, promoterless control) and pGL3-Promoter (Promega, control in assays with pGL3-Prom CD69 3UTR). Plasmid pRL-SV40 (Promega) was used as internal normalization control in all transfections. In settings where transcription factors were overexpressed during the assay, vectors pSG5 VDR (encoding human VDR), pSG5 RXR (encoding human RXR) and pCGN-Smad3 as well as pCGN-Smad4 (encoding human Smad3 and Smad4, respectively) were co-transfected. In corresponding control experiments, the respective empty vectors were transfected in equivalent amounts. One day prior to transfection, cells were seeded in 24-well plates and were transfected using the calcium phosphate method. After 24 h, cells were harvested, lysed and assayed for reporter gene activity using the Dual-Glo® Luciferase Assay system (Promega) according to the manufacturer's protocol. Light emission was determined on a infinite® M200 (Tecan, Switzerland) microplate reader.

Mono Mac 6 and THP-1 were transfected by electroporation using BioRad® Gene Pulser II.

Electroporation of Mono Mac 6 was performed according to Klan *et al.* [20] with some modifications. Cells were grown for 1 d, washed with PBS pH 7.4 and resuspended in RPMI without additives at a density of 4×10^6^ per ml. 300 µl of cell suspension were transferred into a 0.4 cm electroporation cuvette (Invitrogen) and 50 µl of plasmid solution, containing 20 µg reporter plasmid and 1 µg pRL-SV40 (Promega) as internal control, were added. Electroporation was conducted at 975 µF and 200 V. Cuvettes were immediately transferred on ice for 20 minutes. Cells were transferred into 50 ml prewarmed growth medium without phenol red and were treated. Eight hours after treatment cells were harvested, washed with PBS and luciferase assay was performed as described for HeLa cells.

Minor modifications for transfection of THP-1 were applied: Electroporation was conducted in BTXpress Electroporation Solution at 250 V and 950 µF.

Cells were transferred into growth medium immediately after electroporation and luciferase assay was performed 12 h after treatment.

### Western Blotting

Cells were cultivated for 24 h in charcoal-stripped FCS, treated and harvested 24 h after treatment. Then, they were lysed with 1% Triton X-100 in PBS containing protease inhibitors (Complete Mini, Roche) and phosphatase inhibitors (PhosStop, Roche), frozen at −80°C, thawed, homogenized (20 G needle) and centrifuged at 10000 g, 4°C for 10 minutes. Protein concentrations were determined with the Pierce® BCA Protein Assay Kit (Thermo Scientific, Rockford, USA). Readout was performed at 562 nm with an infinite® M200 (Tecan, Switzerland) microplate reader. Samples containing equal amounts of protein were mixed with loading buffer (250 mM Tris-HCl, pH 6.8, 5 mM EDTA, 50% glycerol, 10% SDS, 0.05% bromphenol blue; 10% mercaptoethanol added immediately prior use), heated at 99°C for 5 minutes and loaded onto 10 or 12% SDS polyacrylamide gels.

Gels were blotted on nitrocellulose membranes (Hybond^TM^-C extra, Amersham). Membranes were blocked in a mixture of PBS:Odyssey Blocking buffer (Li-Cor Biosciences) (1∶1) for 1.5 h at room temperature or over night at 4°C. Afterwards, membranes were incubated with primary antibodies (Smad3, Abcam; Actin (I-19), Santa Cruz; p38α (C-20), Santa Cruz; Phospho-p38 MAP Kinase (Thr180/Tyr 182), Cell Signalling) dissolved in PBS:Blocking buffer (1∶1) containing 0.1% Tween 20. Membranes were washed three times with PBS/0.1% Tween 20 and once with PBS alone. Membranes were incubated with secondary antibody (IRdye conjugated Antibodies, Li-Cor Biosciences), washed (see above) and scanned with the Odyssey® infrared imager (Li-Cor Biosciences). Immunoreactive proteins were quantified using Odyssey software (Li-Cor Biosciences) and normalized to actin (Smad3) or p38 (phospho-p38) intensity.

### Lentiviral gene silencing

293T cells were seeded in 24-well plates at a density of 6×10^4^ cells per well. After 24 h, cells were transfected using the calcium phosphate method. Transfection mixes contained 10 µg of MISSION® shRNA TRCN0000330056 plasmid with pLKO.1 – puro backbone (Sigma Aldrich) plus 6.5 µg and 3.5 µg of the packaging plasmids pCMVdR8.91 [21] and pMD2.G (www.tronolab.epfl.ch), respectively. Both packaging plasmids were kindly provided by Dr. Manuel Grez, Georg-Speyer-Haus, Frankfurt, Germany. Four hours after transfection, medium was replaced with 1 ml THP-1 growth medium. Virus production was allowed for 72 h. Then, the supernatant of transfected cells was collected and filtered trough a 0.22 µm filter (Roth, Karlsruhe). Four hours prior to transduction, THP-1 cells were seeded in 24-well plates in growth medium containing protaminsulfate (4 µg/ml, Calbiochem). Each well contained 3 × 10^5^ cells in 0.5 ml medium. Six wells were treated with 50 µl of 293T supernatant, respectively. Supernatant of untransfected 293T was used as control for selection. In order to obtain a negative control cell line, 5 µl Mission non Target shRNA control transduction particles (Sigma Aldrich) were used per well. Plates were centrifuged for 90 minutes at 1000× g, 32°C. Subsequently, medium was added to a final volume of 1 ml in each well and cells were incubated at 37°C, 5% CO_2_ for 3 days.

After incubation, cells from each 6 wells were pooled, counted, washed with PBS pH 7.4 and seeded in fresh medium with 1.5 µg/ml puromycin at a density of 3×10^5^ cells/ml. After two weeks of puromycin selection, cells were used for experiments.

### Reagents

PMA, JNK-Inhibitor XI, (5Z)-7-oxozeaenol and (5Z)-zeaenol were obtained from Calbiochem. 1α,25(OH)_2_D_3_, cycloheximide, actinomycin D, calphostin C, staurosporine, SB431542 and U0126 were purchased from Sigma-Aldrich. SB203580 and PD98059 were purchased from Enzo Life Sciences. Human TGF-β was purified according to Werz *et al*. 1996 [22].

### Statistics

Data are presented as mean + standard deviation (SD), derived from a minimum of three independent experiments. Statistical analysis was conducted using the software Graph Pad Prism, version 5.0 (GraphPad Software, San Diego, California, USA). Significance of differences between two groups was analyzed using two tailed, unpaired t-tests. Data expressed as fold changes relative to a reference group were analyzed using one sample t-tests. Analysis of variance (ANOVA) was performed for significance analysis of more than two groups, combined with multi comparison post tests (Dunnett's or Bonferroni's, as indicated in the respective figure legends).

## Results

### CD69 mRNA expression is induced by TGF-β/1α,25(OH)_2_D_3_ in monocytic cells but not in T- or B-cells

**Figure 1:**
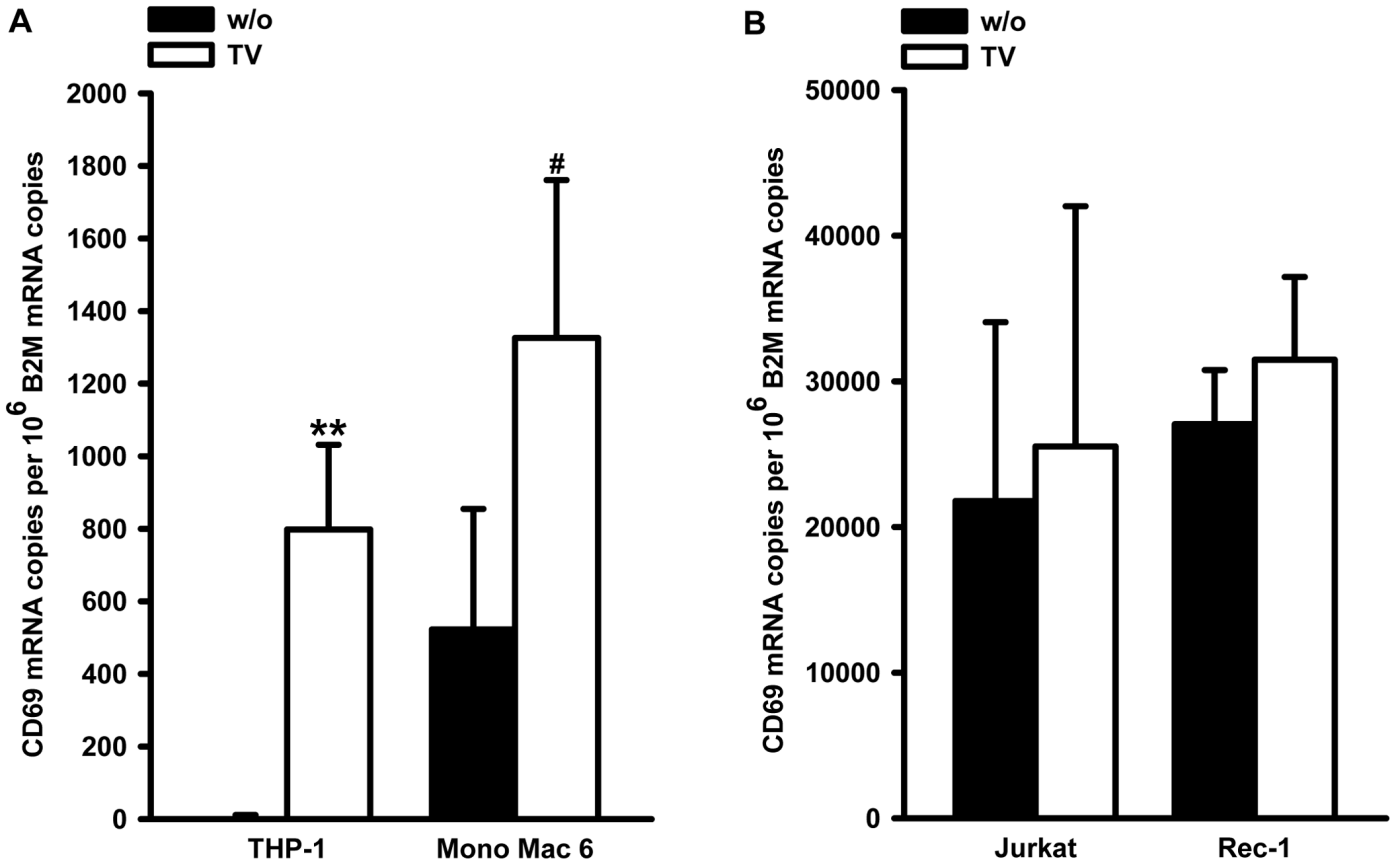
Influence of TGF-β/1α,25(OH)_2_D_3_ on CD69 mRNA expression in different cell types. (A and B) Cells were treated with 1 ng/ml TGF-β and 50 nM 1α,25(OH)_2_D_3_ (TV) for 24 h or left untreated as control (w/o). Subsequently, mRNA levels were determined by qPCR. Statistical analysis was conducted with two tailed, unpaired t-test (*P<0.05; **P<0.01; #P = 0.0638).

Human monocytic cell lines (THP-1 and Mono Mac 6), T-cells (Jurkat) and B-cells (Rec-1) were treated with TGF-β/1α,25(OH)_2_D_3_ for 24 h, and absolute CD69 mRNA levels were determined by RT-qPCR. Almost no CD69 mRNA was detected in untreated THP-1 cells, but mRNA levels could be markedly raised to ∼800 mRNA copies per 10^6^ copies beta-2 microglobulin (B2M) mRNA by treatment with TGF-β/1α,25(OH)_2_D_3_. Slightly higher absolute mRNA levels (∼1300 copies per 10^6^ copies B2M) were obtained in TGF-β/1α,25(OH)_2_D_3_ treated Mono Mac 6 cells, starting from a basal mRNA level of 520 molecules CD69 mRNA per 10^6^ B2M, thus resulting in a 2.5-fold increase (Fig. 1A). In contrast, no induction of CD69 mRNA was found in Jurkat or Rec-1 cells, but the basal levels of ∼22.000 (Jurkat) and ∼27.000 (Rec-1) CD69 mRNA molecules per 10^6^ B2M, were considerably higher in these cell lines (Fig. 1B).

### Induction of CD69 mRNA expression by TGF-β/1α,25(OH)_2_D_3_ occurs rapidly and in a concentration dependent manner

**Figure 2:**
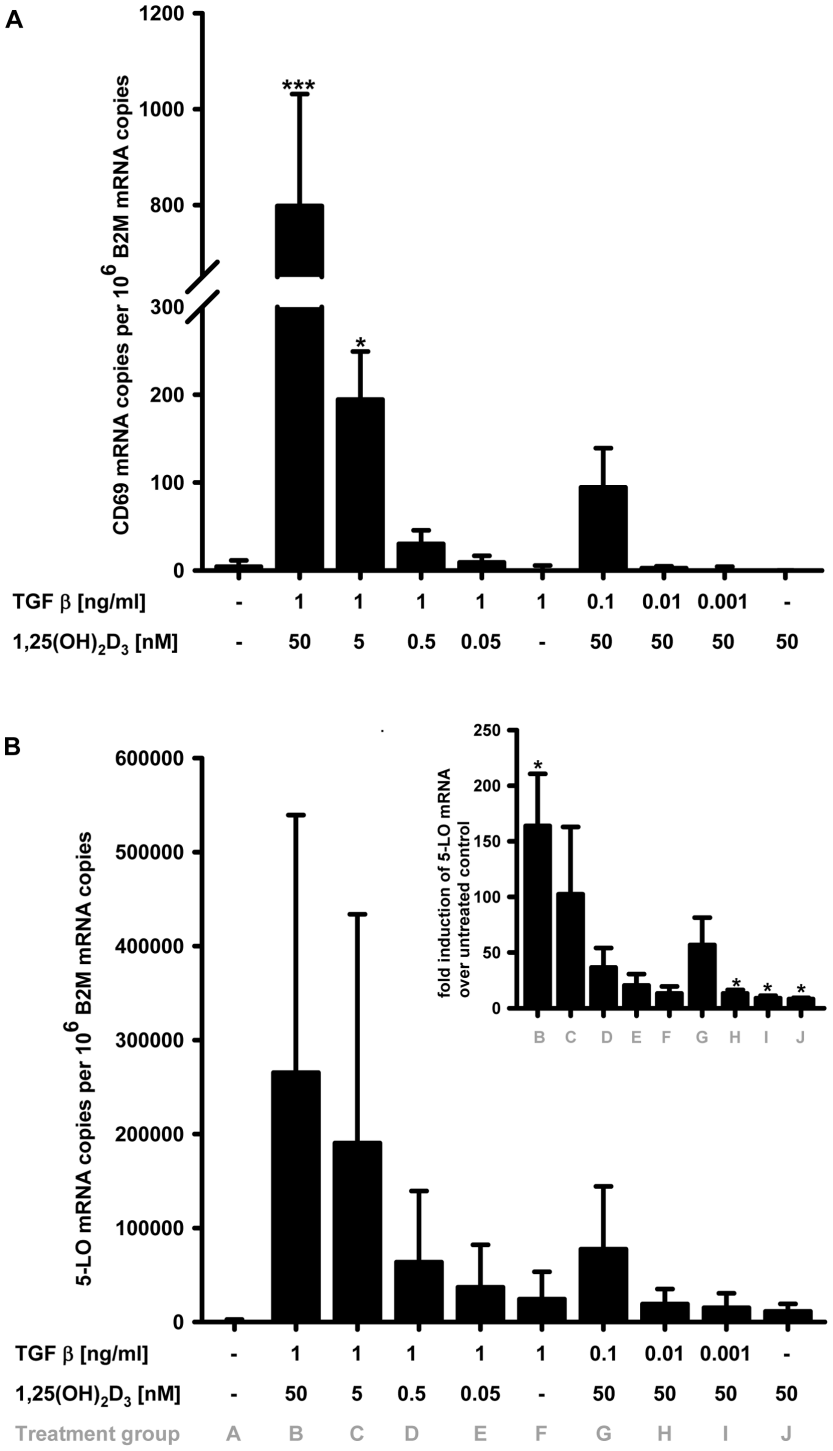
Concentration dependence of CD69 and 5-LO mRNA induction by TGF-β/1α,25(OH)_2_D_3_. (A and B) Cells were treated with varying concentrations of TGF-β and 1α,25(OH)_2_D_3_ and mRNA levels were determined by qPCR. Concentrations, as indicated after “T” for TGF-β and “V” for 1α,25(OH)_2_D_3_, are presented in ng/ml and nM, respectively. The insert in (B) shows fold inductions of 5-LO mRNA over untreated control. One way ANOVA was performed with Dunnett's multiple comparison post test(*P<0.05; ***P<0.001). Fold induction of 5-LO was analyzed with one sample t-test.

To examine the concentration dependence of CD69 mRNA induction, THP-1 cells were either treated with TGF-β 1 ng/ml in combination with 1α,25(OH)_2_D_3_ in concentrations between 50 pM and 50 nM, or conversely with 50 nM 1α,25(OH)_2_D_3_ and TGF-β in varying concentrations from 1 pg/ml to 1 ng/ml. This was compared with single TGF-β (1 ng/ml) or 1α,25(OH)_2_D_3_ (50 nM) treatments, or with untreated controls (Fig. 2A). Neither TGF-β nor 1α,25(OH)_2_D_3_ alone induced CD69 mRNA expression. For the combination of TGF-β and 1α,25(OH)_2_D_3_, a TGF-β concentration of 0.1 ng/ml or higher was required to induce CD69 mRNA expression together with 1α,25(OH)_2_D_3_. When different concentrations of 1α,25(OH)_2_D_3_ were combined with TGF-β 1 ng/ml, 1α,25(OH)_2_D_3_ acted concentration dependently. In contrast, the presence of TGF-β was not a prerequisite for 5-lipoxygenase mRNA induction by 1α,25(OH)_2_D_3_, as 1α,25(OH)_2_D_3_ alone led to a 7.5-fold increase in mRNA expression (Fig. 2B). As observed for CD69, 1α,25(OH)_2_D_3_ also acted concentration dependently on 5-LO mRNA expression in presence of TGF-β in a concentration of 1 ng/ml.

**Figure 3:**
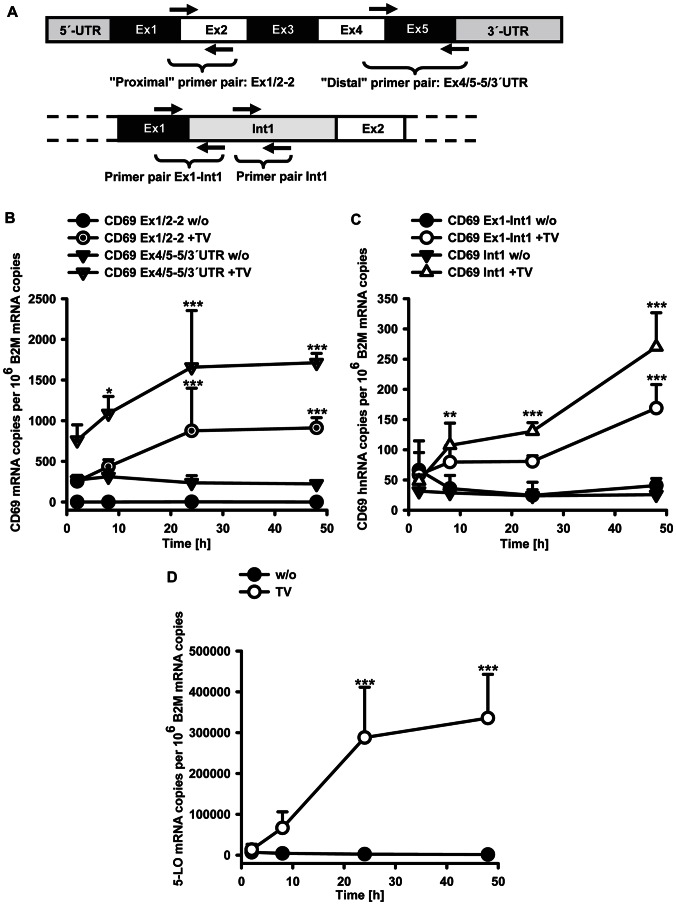
Time dependence of CD69 and 5-LO mRNA induction by TGF-β/1α,25(OH)_2_D_3_. (A) Schematic overview of primer positions on CD69 mRNA (exon and intron sizes are not to scale). (B, C and D) Cells were treated for 2, 8, 24 and 48 h with (TV) or without (w/o) TGF-β/1α,25(OH)_2_D_3_ (1 ng/ml and 50 nM, respectively) and mRNA levels were determined by qPCR. Statistical analysis was done by two way ANOVA and Bonferroni's post test (*P<0.05; **P<0.01; ***P<0.001). The two parts of CD69 gene were analyzed independently. In (B and C), significances as determined by the Bonferroni post test on the data for each part of the gene, compared with the corresponding untreated control, are displayed.

In order to analyze the time course of CD69 mRNA induction, THP-1 cells were treated with TGF-β 1 ng/ml in combination with 1α,25(OH)_2_D_3_ 50 nM for 2, 8, 24 or 48 h. CD69 mRNA abundance was analyzed by RT-qPCR using two primer pairs. Alongside the primer pair specific to the proximal part (exon 1/2-2), which was used for the initial results (Fig. 1–Fig. 2B), an additional primer pair binding to the distal (exon 4/5-5/3′UTR) region of the CD69 gene was employed (Fig. 3A). In comparison to CD69 mRNA levels, 5-LO mRNA expression was monitored. CD69 mRNA expression was induced rapidly, rising from undetectable levels to 250 mRNA copies per 10^6^ B2M mRNAs after 2 h (Fig. 3B). The increase in expression continued almost linearly up to 24 h and reached a plateau afterwards. In contrast, 5-lipoxygenase gene induction was not detectable before 2 h after treatment (Fig. 3D). For transcripts comprising the distal part of the CD69 gene, a basal level of 270 mRNA copies per 10^6^ B2M was detected in untreated cells, which was raised exactly in parallel with the transcripts including the proximal part. In addition, primary CD69 transcripts were quantified using a primer pair specific for the sequence around the junction of exon 1 and intron 1 and one encompassing the first intron of the CD69 gene. The kinetics of the increase in nascent pre-mRNA copy numbers (Fig. 3C) correlated with the increase in mature RNA species (Fig. 3B) with the ratio between the two species averaging at 1∶5. Taken together, the rapid onset of CD69 mRNA induction by TGF-β/1α,25(OH)_2_D_3_, where primary and mature transcripts rise in parallel, indicates that CD69 is a primary TGF-β/1α,25(OH)_2_D_3_ target gene in monocytic cells that is regulated on the level of transcriptional activation by the combination of these compounds.

### Simultaneous presence of TGF-β and 1α,25(OH)_2_D_3_ is required for CD69 and 5-LO mRNA induction

**Figure 4:**
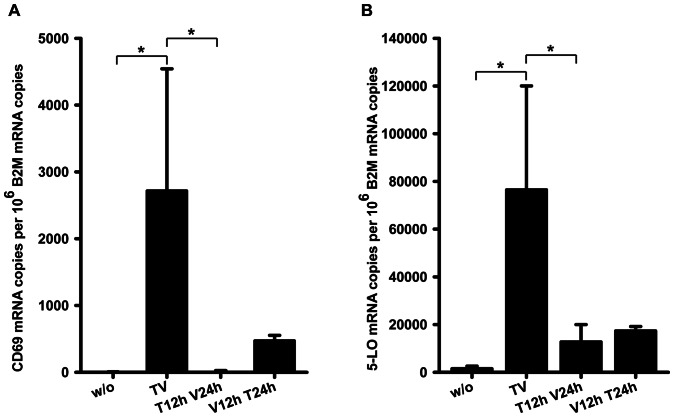
Effects of sequential treatment with TGF-β and 1α,25(OH)_2_D_3_. (A and B) THP-1 were treated with (TV) or without (w/o) 1 ng/ml TGF-β and 50 nM 1α,25(OH)_2_D_3_ for 24 h or pretreated for 12 h with either 1 ng/ml TGF-β (T12h V24h) or 50 nM 1α,25(OH)_2_D_3_ (V12h T24h), washed with PBS and subsequently treated with the respective other compound for another 24 h. CD69 and 5-LO mRNA levels were determined by qPCR. Statistical analysis was conducted with one way ANOVA and Bonferroni's post test (*P<0.05).

In order to investigate whether induction of CD69 and 5-LO mRNA by TGF-β and 1α,25(OH)_2_D_3_ requires the simultaneous presence of both agents, or if one of the agents alone was capable of priming the cells for the action of the other, we performed sequential treatments. For this, we treated THP-1 with either agent alone for 12 h, exchanged medium and incubated with the respective other compound for another 24 h. As controls, we used cells treated with TGF-β/1α,25(OH)_2_D_3_ for 24 h and untreated cells. We found that pre-treatment with neither of the compounds induced mRNA levels to the same extent as simultaneous incubation (Fig. 4). For the CD69 gene, only pre-treatment with 1α,25(OH)_2_D_3_ followed by TGF-β treatment led to modestly enhanced mRNA levels compared to untreated control (Fig. 4A). In contrast, levels of 5-LO mRNA were increased moderately in both ways of pre-treatment as compared to untreated cells (Fig. 4B), however the extent of induction was only slightly higher than that obtained by incubation with the single compounds as observed in the concentration series (Fig. 2B). Thus, combined treatment is necessary for strong induction of both mRNA species and pre-treatment more or less reflects the setting of single treatment.

### CD69 promoter fragments are not responsive for activation by TGF-β/1α,25(OH)_2_D_3_

**Figure 5:**
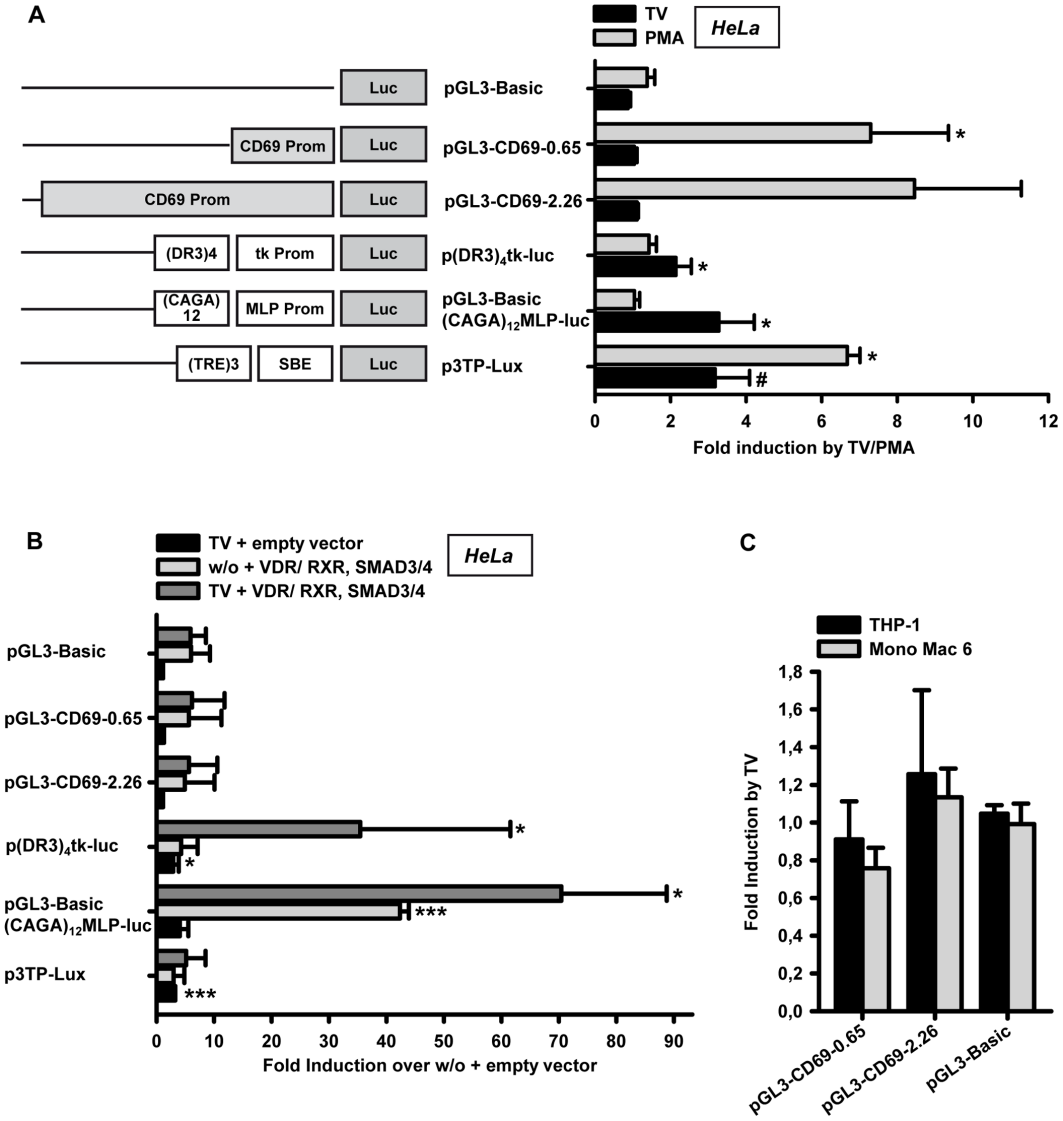
Reporter gene analysis of the proximal CD69 promoter. (A) HeLa cells were transfected with the indicated reporter plasmids and treated with or without 1 ng/ml TGF-β and 50 nM 1α,25(OH)_2_D_3_ (TV) or 15 ng/ml phorbol 12-myristate 13-acetate (PMA). Luciferase activity was measured 24 h after treatment as described under “Materials and Methods”. The values are expressed as fold induction over untreated cells. Statistical analysis was performed with one sample t-test (*P<0.05; # = 0.0538). (B) HeLa cells were transfected with the indicated reporter plasmids and expression vectors encoding human vitamin D receptor (VDR), retinoid X receptor (RXR), Smad3 and Smad4 or the corresponding empty vectors. Cells were either treated with 1 ng/ml TGF-β and 50 nM 1α,25(OH)_2_D_3_ (TV) or left untreated (w/o). Luciferase activity was measured 24 h after treatment. The values are expressed as fold inductions over untreated cells cotransfected with empty vectors. Statistical analysis was conducted with one sample t-test (*P<0.05; ***P<0.001). (C) THP-1 or Mono Mac 6 cells were transfected with the indicated reporter plasmids and were treated with or without 1 ng/ml TGF-β and 50 nM 1α,25(OH)_2_D_3_ (TV). After 12 h (THP-1) and 8 h (Mono Mac 6), luciferase assay was performed. The values are expressed as fold induction over untreated cells.

We analyzed activation of the CD69 promoter region by TGF-β/1α,25(OH)_2_D_3_ in HeLa, THP-1 and Mono Mac 6 cells using transient reporter gene assays. The CD69 promoter constructs comprised −0.65 to +0.08 or −2.26 to +0.08 kb of the promoter region (relative to the transcription start site) in front of the luciferase reporter gene. These regions have been characterized as the most responsive parts of the CD69 promoter to PMA activation [23]. Transfected cells were treated with TGF-β/1α,25(OH)_2_D_3_ for 24 h before luciferase activation. No induction by TGF-β/1α,25(OH)_2_D_3_ was detected for the CD69 promoter constructs in HeLa cells, whereas PMA activated both fragments approximately 8-fold. Reporter constructs serving as positive control for TGF-β mediated promoter activation via Smads (pGL3-Basic-(CAGA)_12_-MLP-Luc) or containing vitamin D response elements (p(DR3)_4_tk luc) were activated 3-fold and 2-fold by TGF-β/1α,25(OH)_2_D_3_, respectively, but not by PMA. The positive control construct p3TP-Lux, which contains adjacent SBEs and TPA/PMA responsive elements (TREs), was activated 3-fold by TGF-β/1α,25(OH)_2_D_3_ and 7-fold by PMA (Fig. 5A).

Next, the reporter constructs were either cotransfected with expression vectors encoding the transcription factors vitamin D receptor (VDR), retinoid X receptor (RXR), Smad3 and Smad4, or with empty vectors. Luciferase activity was increased for all constructs by coexpression of the transcription factors, and the inducibility of the control constructs for (1α,25(OH)_2_D_3_) and TGF-β response increased dramatically, but induction of CD69 promoter constructs by TGF-β/1α,25(OH)_2_D_3_ was still not detectable (Fig. 5B). Similarly, CD69 promoter constructs did not respond when THP-1 and Mono Mac 6 as monocytic cells were transfected (Fig. 5C).

From this, we concluded that (1) regulatory elements located outside the CD69 proximal promoter regions might be responsible or needed additionally in order to activate CD69 mRNA transcription (2) complex chromatin remodelling events, that can not be properly reproduced in transient reporter assays might be involved in TGF-β/1α,25(OH)_2_D_3_ action, or (3) that TGF-β/1α,25(OH)_2_D_3_ treatment acts on CD69 mRNA stability.

### CD69 mRNA stability is not influenced by TGF-β/1α,25(OH)_2_D_3_

**Figure 6:**
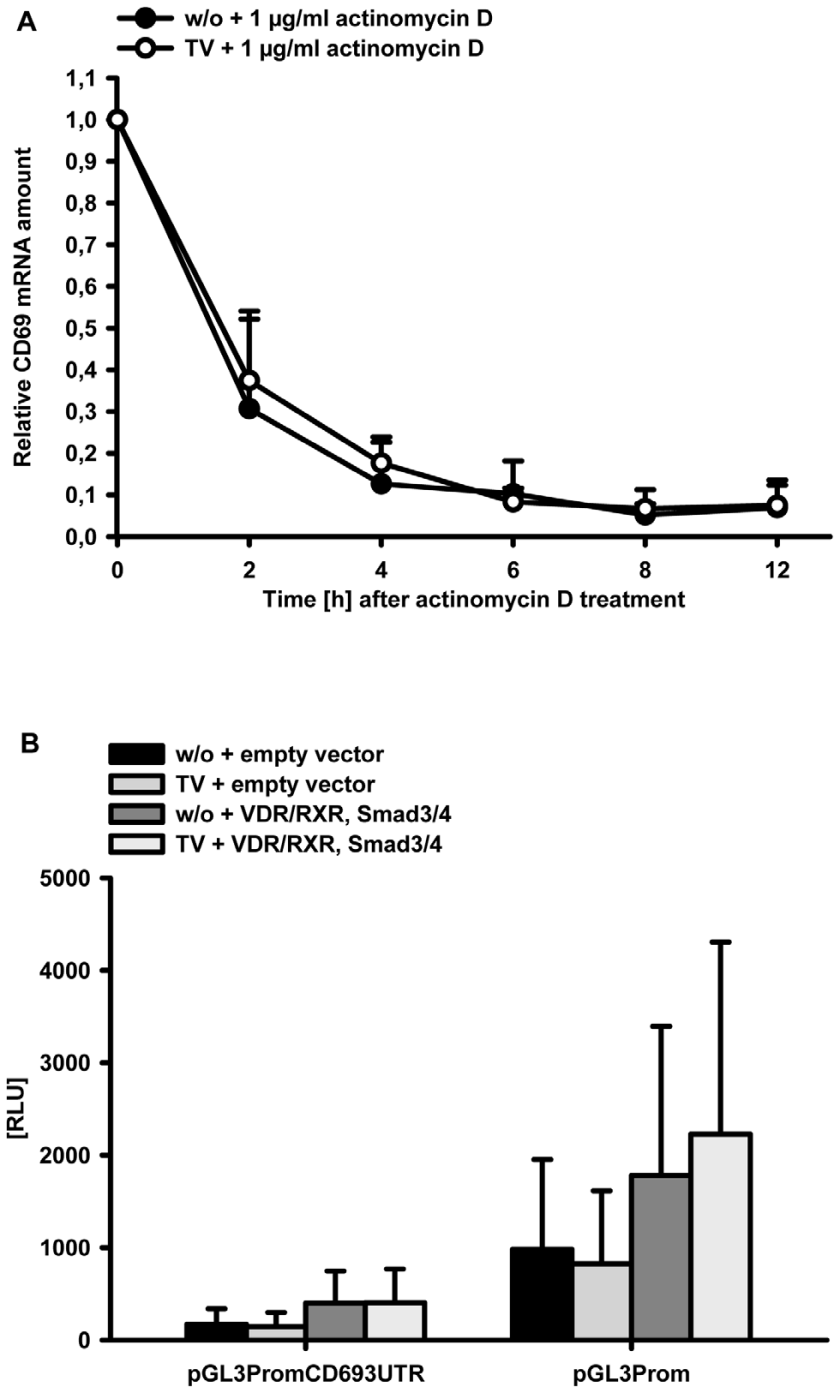
Influence of TGF-β/1α,25(OH)_2_D_3_ on CD69 mRNA half life. (A) Cells were treated with 1 ng/ml TGF-β and 50 nM 1α,25(OH)_2_D_3_ (TV) for 24 h or left untreated as control (w/o). Then 1 µg/ml actinomycin D was added to both treatment groups and cells were harvested at the indicated time points after actinomycin D treatment. Subsequently, mRNA levels were determined by qPCR. Data are expressed relative to time zero (immediately before addition of actinomycin D) (B) HeLa cells were transfected with the indicated reporter plasmids and expression vectors encoding human vitamin D receptor (VDR), retinoid X receptor (RXR), Smad3 and Smad4 or the corresponding empty vectors. Cells were either treated with 1 ng/ml TGF-β and 50 nM 1α,25(OH)_2_D_3_ (TV) or left untreated (w/o). Luciferase activity was measured 24 h after treatment. The data are presented as relative luminescence units (RLU, luciferase activity of reporter plasmid normalized to the luciferase activity of the internal standard plasmid).

In order to assess whether the combination of TGF-β and 1α,25(OH)_2_D_3_ acts on CD69 mRNA stability, qRT-PCR analysis was carried out after incubation with the transcription inhibitor actinomycin D. As THP-1 cells were not suitable for this approach due to their lack of basal CD69 mRNA expression, Mono Mac 6 cells were used. Mono Mac 6 cells were preincubated with TGF-β/1α,25(OH)_2_D_3_ or left untreated for 24 h. Then 1 µg/ml actinomycin D was added and CD69 mRNA level was analyzed at the indicated time points. CD69 mRNA half life was comparable (less than 2 h) between treated and untreated samples (Fig. 6A).

The 3′UTR region of the CD69 gene is known to contain destabilizing AU-rich elements [24], and stabilization of mRNAs containing AU-rich elements by TGF-β has been shown [25]. Therefore, we inserted the CD69 3′UTR into a SV40-promoter driven luciferase reporter vector and analyzed changes in relative luciferase activity as compared to the empty vector with or without TGF-β/1α,25(OH)_2_D_3_ treatment. As expected, insertion of the CD69 3′UTR decreased luciferase activity 5.8-fold, but this effect could not be reverted by TGF-β/1α,25(OH)_2_D_3_ (Fig. 6B).

Thus, the stability of CD69 mRNA is not influenced by TGF-β/1α,25(OH)_2_D_3_ treatment.

### Induction of CD69 mRNA expression by TGF-β/1α,25(OH)_2_D_3_ depends on Smad3

**Figure 7:**
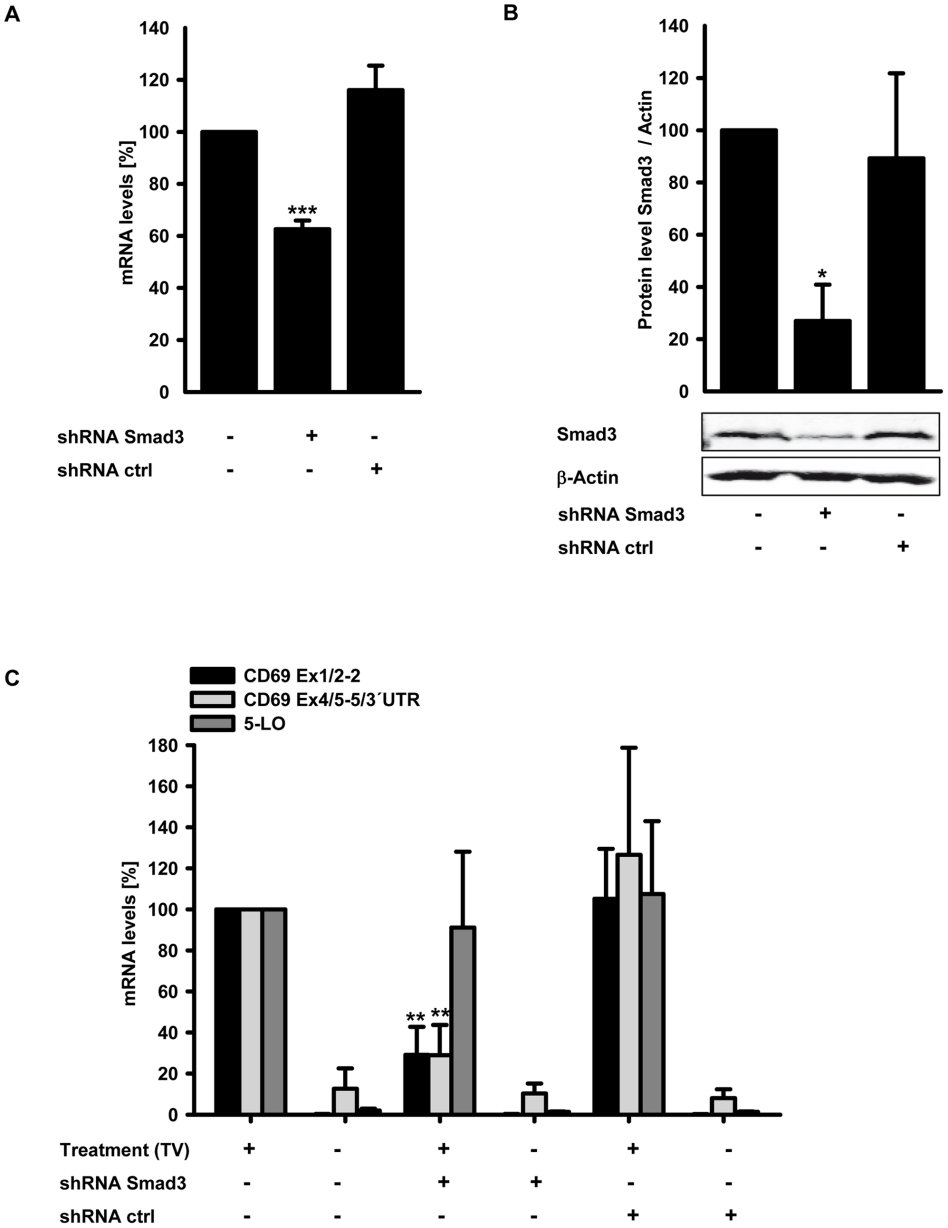
Effects of Smad3 gene silencing on CD69 and 5-LO gene regulation by TGF-β/1α,25(OH)_2_D_3_. (A) Smad3 mRNA copy numbers of untransduced THP-1, THP-1 transduced with shRNA against Smad 3 and THP-1 transduced with non target shRNA were determined by qPCR. Smad 3 mRNA levels are displayed relative to untransduced THP-1 cells. (B) Western Blot analysis of Smad3 protein in untransduced THP-1, THP-1 transduced with shRNA against Smad3 and THP-1 transduced with non target shRNA. Protein level is displayed relative to untransduced THP-1. (C) qPCR analysis of CD69 and 5-LO mRNA levels in untransduced THP-1, THP-1 transduced with shRNA against Smad3 and THP-1 transduced with non target shRNA with and without treatment with 1 ng/ml TGF-β and 50 nM 1α,25(OH)_2_D_3_ (TV) for 24 h, respectively. Results are presented relative to treated untransduced THP-1. For statistical analysis of (A), (B) and (C) one sample t test was used (*P<0.05; **P<0.01; ***P<0.001).

Next, we analyzed whether TGF-β contributes to mRNA upregulation via the classical Smad pathway. For this purpose, we employed lentiviral shRNA for functional knockdown of Smad3 expression in THP-1 cells. At first, five independent shRNA constructs were analyzed for Smad3 mRNA reduction by transient transfection of 293T cells, and the most effective construct was subsequently used to generate a stable cell line (data not shown). A negative control cell line was established using non target shRNA. The Smad3 shRNA construct led to a 37% and 73% reduction in mRNA and protein level, respectively, compared to untransduced cells (Fig. 7A and B).

The cells were incubated with TGF-β/1α,25(OH)_2_D_3_ for 24 h, and CD69 and 5-LO mRNA levels were analyzed by RT-qPCR. Smad knockdown resulted in a 71% decrease of CD69 mRNA induction by TGF-β/1α,25(OH)_2_D_3_. In contrast, induction of 5-LO mRNA was not altered significantly (Fig. 7C). Therefore, we clearly demonstrate that CD69 mRNA induction by TGF-β/1α,25(OH)_2_D_3_ depends on Smad3, whereas 5-LO upregulation seems to be independent of Smad3.

### Induction of CD69 mRNA expression by TGF-β/1α,25(OH)_2_D_3_ is mediated by the TGF-β receptor and is blocked by inhibitors of the MAPK pathway

In order to dissect the mechanism of TGF-β/1α,25(OH)_2_D_3_ on CD69 and 5-LO gene expression in more detail, THP-1 cells were treated with TGF-β/1α,25(OH)_2_D_3_ for 24 h in presence or absence of different inhibitors before CD69 or 5-LO mRNA levels were determined. The inhibitors were directed against proteins that are either implicated in TGF-β signalling (TGF-β receptor I; p38 MAPK, Mek/Erk and Jnk; TGF-β activated kinase) or can be connected to CD69 promoter activation (PKC). The MAPK pathways p38 MAPK, Mek/Erk, Jnk and TGF-β activated kinase are major routes of non-Smad pathways of TGF-β signalling [11], [12], and PKC inhibitors were employed due to the fact that the proximal CD69 promoter is known to contain AP1 binding sites [26] and is responsive to phorbol ester stimulation [23]. Moreover, AP1 is a known interaction partner of activated Smad3/4 as TGF-β effector proteins [27].

**Figure 8:**
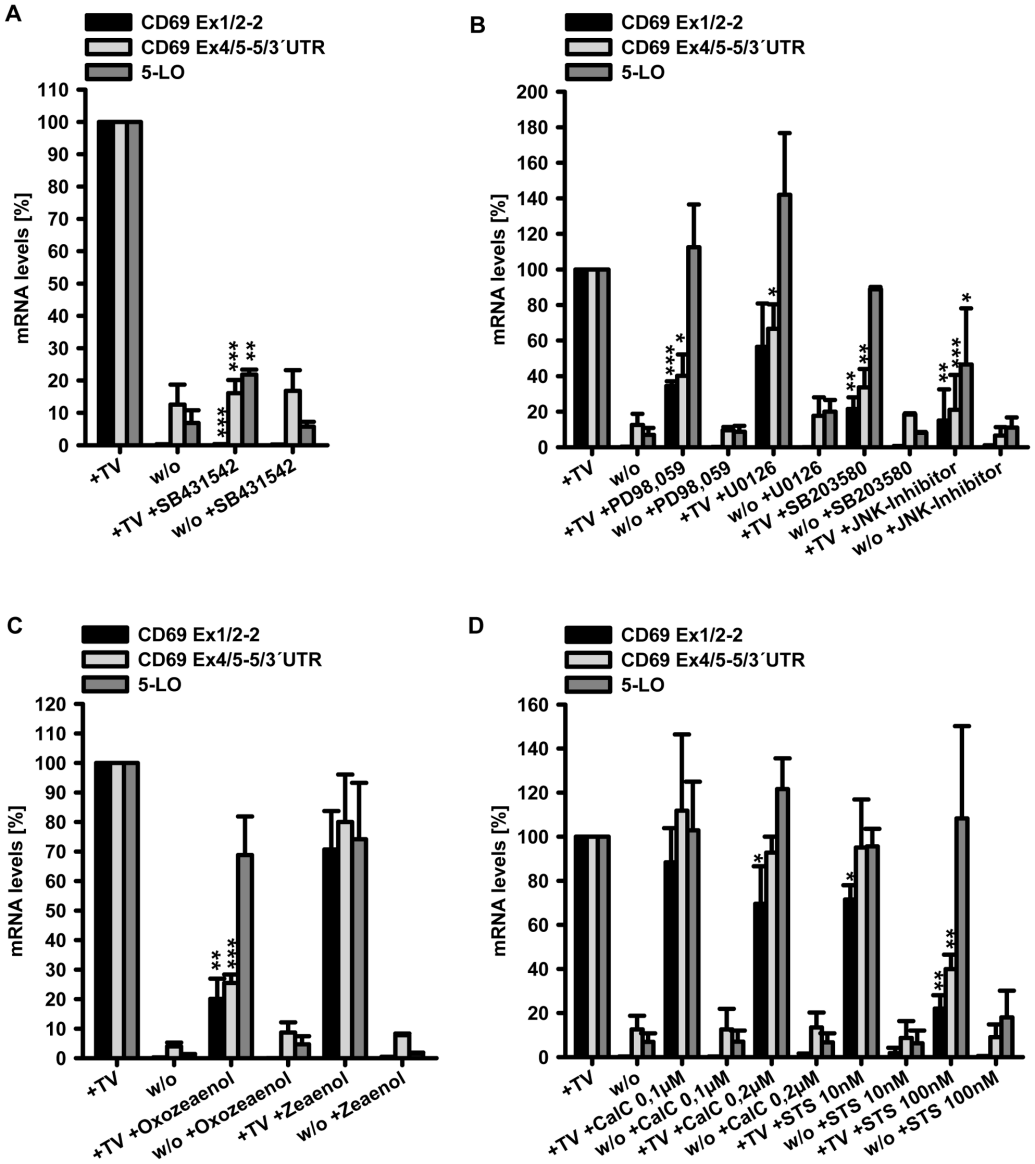
Inhibitor studies on signalling mechanisms of TGF-β/1α,25(OH)_2_D_3_-mediated CD69 and 5-LO gene regulation. (A–D) THP-1 cells were treated with 1 ng/ml TGF-β and 50 nM 1α,25(OH)_2_D_3_ (TV) or left untreated (w/o) for 24 h after 30 min pre-incubation with or without the indicated inhibitors. Inhibitor concentrations used were as follows: SB431542 10 µM, PD98059 50 µM, U0126 10 µM, SB203580 10 µM, JNK-Inhibitor XI 10 µM as well as oxozeaenol and zeaenol 0.3 µM, respectively. Applied concentrations for calphostin C (CalC) and staurosporine (STS) are indicated. Levels of mRNA are presented relative to the corresponding values after TGF-β/1α,25(OH)_2_D_3_ treatment. Statistical analysis was conducted with one sample t test (*P<0.05; **P<0.01; ***P<0.001).

We found that the TGF-β I receptor kinase antagonist SB431542 completely blocked CD69 mRNA induction by TGF-β/1α,25(OH)_2_D_3_ and inhibited 5-LO mRNA induction by 78%, which clearly shows that TGF-β/1α,25(OH)_2_D_3_ completely (CD69) or mainly (5-LO) act via specific, receptor-mediated effects (Fig. 8A). The increase in proximal CD69 mRNA was diminished after treatment with inhibitors against the three major MAPK pathways p38 MAPK (SB203580), Erk (PD98059, U0126) and Jnk (Jnk Inhibitor XI), by 78%, 44–66% and 85%, respectively. In contrast, 5-lipoxygenase induction was only affected by Jnk inhibition (54% reduction), whereas p38 MAPK and Erk inhibition had only little effects (Fig. 8B). Inhibition of TGF-β activated kinase 1 (TAK1) led to a 80% decrease of CD69 mRNA induction, as compared to 31% for the 5-LO gene. Zeaenol, which served as negative control compound for the selective TAK1 inhibitor Oxozeaenol, did not significantly change CD69 or 5-LO mRNA expression (Fig. 8C).

With the PKC inhibitors calphostin C and staurosporine, we found that neither calphostin C at 0.1 or 0.2 µM nor staurosporine 10 nM potently blocked CD69 mRNA induction, only at 100 nM staurosporine a 60 and 78% reduction was detectable for proximal and distal parts of CD69 mRNA, respectively. 5-LO mRNA abundance was not affected by both inhibitors (Fig. 8D).

Taken together, TGF-β/1α,25(OH)_2_D_3_ act differently on CD69 and 5-LO mRNA regulation regarding the involvement of the MAPK pathway, with inhibition being prominently detected for the CD69 gene. It is unlikely that PKC plays a major role in the regulation of CD69 gene expression by TGF-β/1α,25(OH)_2_D_3_ in monocytic cells.

### TGF-β/1α,25(OH)_2_D_3_ induce p38 phosphorylation that is blocked by TAK1 inhibition

**Figure 9:**
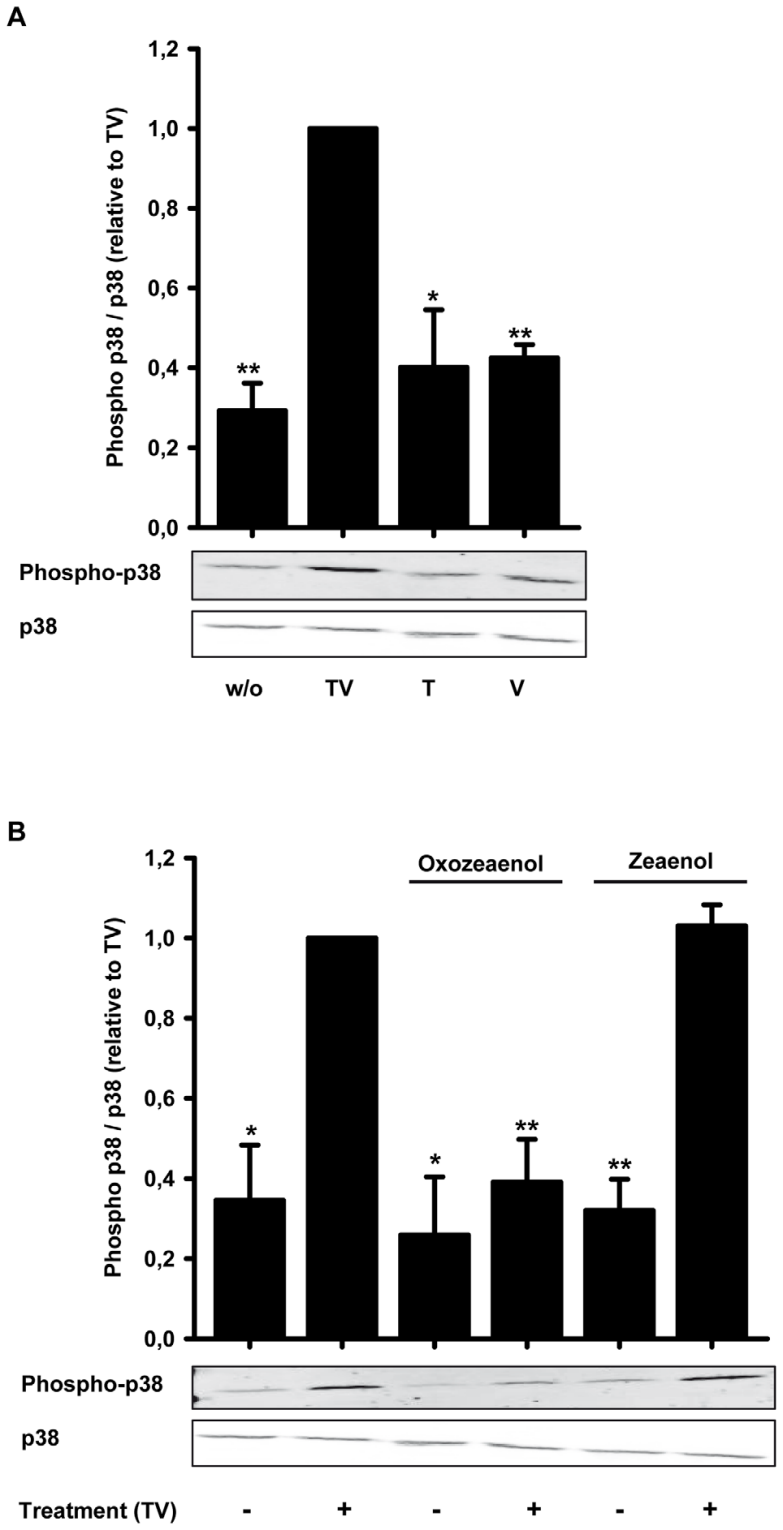
Western Blot analysis of p38 phosphorylation in dependence of treatment with TGF-β and/or 1α,25(OH)_2_D_3_. (A) Western Blot analysis of p38 protein and phosphorylated p38 protein in THP-1 incubated for 24 h with 1 ng/ml TGF-β (T), 50 nM 1α,25(OH)_2_D_3_ (V), both agents (TV) or without treatment (w/o). The relative phospho-p38 level (normalized to total p38 protein) of TGF-β/1α,25(OH)_2_D_3_ treated cells was set to 1. (B) THP-1 cells were treated with (TV) or without (w/o) 1 ng/ml TGF-β and 50 nM 1α,25(OH)_2_D_3_ for 24 h after 30 min pre-incubation with or without the indicated inhibitors. Applied concentrations of oxozeaenol and zeaenol were 0.3 µM, respectively. Phosphorylated p38 compared to whole p38 protein was analyzed by western blot. Relative protein amount of TGF-β/1α,25(OH)_2_D_3_ treated cells was set to 1. Statistical analysis of (A) and (B) was done with one sample t-test (*P<0.05; **P<0.01).

Our inhibitor studies revealed that inhibitors of TAK1 and p38 MAPK reduced CD69 mRNA induction to exactly the same extent (Fig. 8B–C). This is in line with findings that activation of p38 by TGF-β is mediated by TAK1 in HEK293 cells [28]. Therefore, we first analyzed whether TGF-β/1α,25(OH)_2_D_3_ activate p38 MAPK by phosphorylation in THP-1 cells. Interestingly, we found that only the combination of TGF-β and 1α,25(OH)_2_D_3_, but not the single compounds, led to prominent p38 phosphorylation (Fig. 9A). Subsequently, we tested whether TAK1 inhibition abrogates p38 phosphorylation using the TAK1 inhibitor oxozeaenol and the control compound zeaenol in TGF-β/1α,25(OH)_2_D_3_ treated THP-1 cells. Oxozeaenol almost completely inhibited p38 phosphorylation, whereas zeaenol had no effect on the phosphorylation status (Fig. 9B).

These findings show that TGF-β/1α,25(OH)_2_D_3_ cooperatively induce TAK1-mediated p38 phosphorylation.

### Combined effects of TGF-β/1α,25(OH)_2_D_3_ and PMA on CD69 mRNA induction revert over time

**Figure 10:**
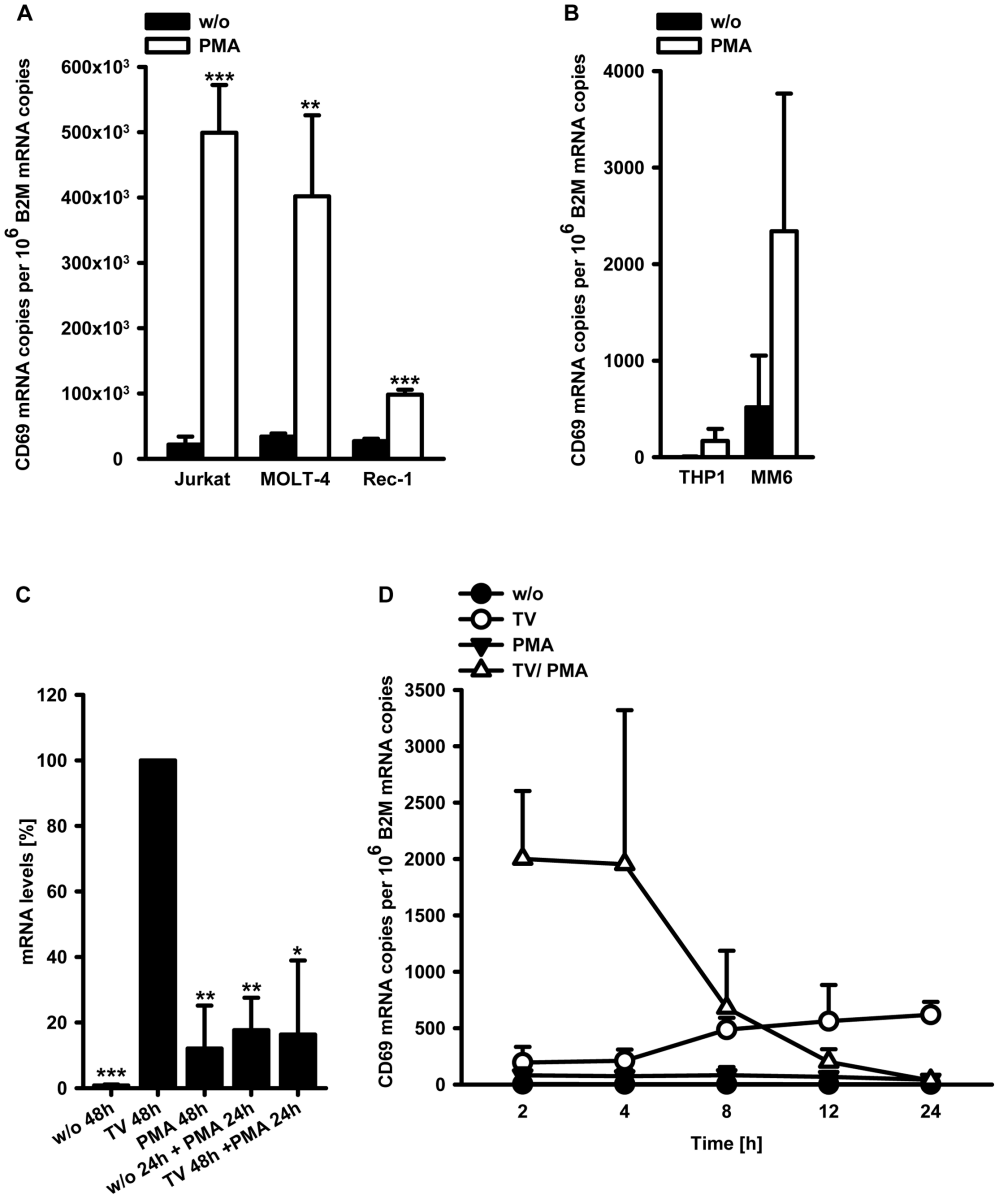
Influence of PMA on CD69 mRNA levels in dependence of time and cotreatment. (A and B) Cells were treated with (PMA) or without (w/o) 20 ng/ml PMA for 24 h. Subsequently, mRNA levels were determined by qPCR. Statistical analysis of (A) and (B) was conducted with unpaired, two-tailed t-test (**P<0.01; ***P<0.001). (C) THP-1 were treated with 1 ng/ml TGF-β and 50 nM 1α,25(OH)_2_D_3_ (TV), 20 ng/ml PMA (PMA) or left untreteated as control (w/o) for 48 h. Alternatively, cells were pretreated with (TV) or without (w/o) 1 ng/ml TGF-β and 50 nM 1α,25(OH)_2_D_3_ for 24 h and 20 ng/ml PMA was added for further 24 h. Subsequently, CD69 mRNA levels were determined by qPCR. The CD69 mRNA level of cells treated with TGF-β/1α,25(OH)_2_D_3_ for 48 h was set to 100. Statistical analysis was conducted with one sample t-test (*P<0.05; **P<0.01; ***P<0.001). (D) THP-1 were incubated with 1 ng/ml TGF-β and 50 nM 1α,25(OH)_2_D_3_ (TV), 20 ng/ml PMA (PMA), all three agents together (TV/PMA) or left untreated as control (w/o) for the indicated times. Subsequently, CD69 mRNA levels were determined by qPCR.

In lymphocytes, CD69 expression has been studied extensively, and it is well known that the almost undetectable CD69 mRNA levels in these cells are induced strongly upon PMA treatment [23], [29], [30], [31]. We determined absolute CD69 mRNA levels in PMA treated Jurkat, MOLT-4 and Rec-1 cells and observed several hundred-fold higher mRNA levels compared to those in TGF-β/1α,25(OH)_2_D_3_ treated monocytic cells (Fig. 10A; Fig. 1A). Analogously, PMA treatment of monocytic cells led to significantly lower CD69 mRNA levels than in equally stimulated lymphocytes (Fig. 10B). In Mono Mac 6 cells, CD69 mRNA levels were slightly higher after PMA treatment compared to incubation with TGF-β and 1α,25(OH)_2_D_3_, whereas in THP-1 cells TGF-β/1α,25(OH)_2_D_3_ treatment led to 4.8 fold higher CD69 mRNA levels than PMA treatment (Fig. 10B; Fig. 1A).

We investigated the possibility that TGF-β/1α,25(OH)_2_D_3_ act on the CD69 gene locus of monocytic cells by converting the chromatin architecture into a permissive state and therefore prime cells for PMA response. To elucidate this, we treated THP-1 cells with TGF-β/1α,25(OH)_2_D_3_ or PMA alone for 48 h, or cells were sequentially incubated with or without TGF-β/1α,25(OH)_2_D_3_ for 24 h, and PMA was added for further 24 h.

Unexpectedly, instead of acting cooperatively, PMA efficiently inhibited CD69 mRNA induction by TGF-β/1α,25(OH)_2_D_3_ in this setting (Fig. 10C).

For further analysis, we performed a time course experiment with combined treatment of TGF-β/1α,25(OH)_2_D_3_ and PMA, as well as either treatment alone, or no treatment (Fig. 10D). Remarkably, we observed a strong synergism of TGF-β/1α,25(OH)_2_D_3_ and PMA after 2 and 4 h, whereas PMA abolished the effect of TGF-β/1α,25(OH)_2_D_3_ after 12 and 24 h of treatment.

## Discussion

CD69 has been functionally linked to 5-lipoxygenase, the key enzyme in leukotriene biosynthesis, in a study with CD14-positive monocytes [3]. Both CD14 and 5-lipoxygenase are known target genes for TGF-β and 1α,25(OH)_2_D_3_ in this cell type [7], [8], [32], [33]. In contrast, at present no data are available on the mechanism of CD69 gene regulation in monocytic cells.

Here, we demonstrate that CD69 is a novel TGF-β/1α,25(OH)_2_D_3_ target gene and is therefore coexpressed with 5-lipoxygenase, but that individual mechanisms account for TGF-β/1α,25(OH)_2_D_3_ action regarding the two genes.

We could find that CD69 mRNA upregulation by TGF-β/1α,25(OH)_2_D_3_ occurs cell-specifically in monocytes, but not in T- or B-cells. This expression pattern conforms to the 5-LO gene, where TGF-β/1α,25(OH)_2_D_3_ do not induce expression in T- and B-cells either (Dieter Steinhilber, unpublished data). It must however be noted as a difference between the two genes that T-cells do express CD69 upon cellular activation by different signalling molecules [29], but are 5-LO negative cells [34]. The similarities in the expression pattern could indicate that the proteins have a monocyte-specific functional relationship.

The two monocytic cell lines THP-1 and Mono Mac 6 differed in the occurrence of basal CD69 mRNA expression; no CD69 mRNA could be detected in untreated THP-1 cells whereas Mono Mac 6 cells exhibited a basal level of CD69 mRNA. It has been shown that these two cell lines diverge in their differentiation status, with Mono Mac 6 being more differentiated towards mature monocytes than THP-1 [35]. Therefore, a basal level of CD69 expression is possibly acquired during monocyte maturation.

We observed marked differences regarding CD69 and 5-lipoxygenase mRNA induction by TGF-β/1α,25(OH)_2_D_3_. In both cases, the combination of TGF-β and 1α,25(OH)_2_D_3_ was required for the maximum effect, but in case of 5-LO also 1α,25(OH)_2_D_3_ alone was sufficient to induce mRNA expression, whereas CD69 expression strictly depends on the presence of TGF-β (Fig. 2A and B). This is also reflected by the results of sequential treatment (Fig. 4). The results show that cooperation between TGF-β/1α,25(OH)_2_D_3_ depends on simultaneous presence of both agents and neither of the agents alone is capable of priming for the full effect. Therefore, for both genes, combined action of TGF-β and 1α,25(OH)_2_D_3_ is rather a primary than a secondary effect. Furthermore, we detected kinetic differences between the two genes. CD69 mRNA abundance was induced within 2 h, and thus increased more rapidly than 5-LO mRNA levels, where a significant induction could only be observed later than 2 h. The rapid onset of CD69 mRNA induction also suggests that CD69 is a primary TGF-β/1α,25(OH)_2_D_3_ target gene. Methodologically, primary and secondary target genes are often distinguished by the use of protein synthesis inhibitors like cycloheximide. However, due to the fact that the CD69 3′UTR contains destabilizing AU-rich elements [24], and such mRNAs are known to be strongly stabilized by cycloheximide [36], this approach was not suitable for the CD69 gene. On the other hand, TGF-β has been shown to increase the half-life of mRNAs which contain AU-rich elements in their 3′UTRs [25], [37]. Therefore, we investigated whether CD69 mRNA induction by TGF-β/1α,25(OH)_2_D_3_ is based on mRNA stabilization. However, neither half life studies using the transcription inhibitor actinomycin D nor 3′UTR analysis using reporter genes indicated an influence of TGF-β/1α,25(OH)_2_D_3_ on CD69 mRNA stability. Analysis of pre-mRNA species revealed that the combination of TGF-β and 1α,25(OH)_2_D_3_ directly activates CD69 transcription (Fig. 3C). Regarding the quantification of mature mRNA species over time, we observed that transcripts including the proximal (exons 1–2) and the distal (exon 4–3′UTR) part of the CD69 gene increased in parallel upon TGF-β/1α,25(OH)_2_D_3_ treatment, however, the number of transcripts comprising the distal part was uniformly higher for all time points, for both treated and untreated cells (Fig. 3B). Several reasons could possibly account for this observation. It is conceivable that a constantly expressed transcript comprising the distal part originates from a second gene that overlaps with the CD69 gene, or from an alternative transcription start site within the distal part of the CD69 gene. Moreover, alternatively spliced transcripts lacking proximal parts, as described for the porcine CD69 gene [38], come into consideration. However, such an overlapping gene, or such transcripts, respectively, have not been described as yet. Furthermore, slow degradation of CD69 mRNA starting from the 5′-end may render species that only comprise the distal part.

To further analyze the mechanisms of TGF-β/1α,25(OH)_2_D_3_ action, we used (a) lentivirus-delivered stable gene silencing of Smad3 as the central component of Smad-dependent TGF-β signalling, (b) pharmacological inhibitors, (c) reporter gene assays with constructs containing CD69 promoter fragments of different length.

First, we could clearly show by stable silencing of Smad3 expression in THP-1 cells that TGF-β/1α,25(OH)_2_D_3_-mediated CD69 induction depends on Smad3, whereas this is not the case for 5-LO. Regarding 5-LO expression, this is in contrast to findings that overexpression of Smads3/4 induces plasmid-based 5-lipoxygenase expression in reporter gene assays [39]. These differences may be attributed to the fact that the reporter gene assay was conducted in a heterologous cell system and that a reporter construct does not reflect the genomic situation in a 5-LO positive cell as it was used in our approach. Using pharmacological inhibitors, we could clearly show for CD69 that the TGF-β/1α,25(OH)_2_D_3_ signal completely depends on stimulation of a specific receptor, the TGF-β receptor I. Subsequently, we used inhibitors of TAK1 and the three major MAPK pathways p38 MAPK, Jnk and Erk. Our data reveal that the classical MAPKs and TAK1 play a role in CD69 regulation by TGF-β/1α,25(OH)_2_D_3_. Interestingly, TAK1 and p38 MAPK inhibition diminished CD69 induction to the same extent, which pointed to a direct dependence between these two kinases, as demonstrated for TGF-β treated HEK293 cells [28]. This interplay could indeed be confirmed by Western Blot analysis of differences in p38 phosphorylation in the presence or absence of TAK1 inhibitor. Taken together, this suggests that TGF-β/1α,25(OH)_2_D_3_ act on CD69 mRNA expression via TAK1-mediated p38 activation.

Regarding 5-LO regulation and MAPK involvement, we only found 50% inhibition by the Jnk inhibitor. The 5-LO promoter contains two AP1 response elements, at pos. −2990 to −2984 (TGTCTCA [40]) and at pos. −3246 to −3240 (TGGCTCA [41]) from the translational start site, which could potentially be addressed by activated c-Jun. However, no activation by TGF-β/1α,25(OH)_2_D_3_ of 5-LO promoter fragments containing these elements could be seen in transient reporter assays [42]. We obtained the same negative result for CD69 promoter fragments (Fig. 5), which contain AP1 sequences near the transcription start site [23]. It is possible that Jnk-mediated protein activation, classically of c-Jun, is not sufficient to address the AP1 elements in the promoters without activation of interacting proteins like c-fos, which is not a Jnk substrate [43]. Jnk might therefore act posttranscriptionally on CD69 and 5-LO mRNA expression. It is also possible that further proteins are needed in the multiprotein complex interacting with the general transcriptional machinery that are not present under the circumstances of transient reporter assays.

The reporter constructs that were used to analyze the effect of TGF-β/1α,25(OH)_2_D_3_ on the CD69 promoter included the sequences between positions −0.65 to +0.08 kb and −2.26 to +0.08 kb of the CD69 gene (relative to the transcription start site), respectively. The shorter construct contains two putative vitamin D response elements and two additional potential VDREs are located in the longer construct, as predicted by the *in silico* promoter analysis program “NHR Scan” [44]. Five putative Smad binding elements (SBE) are located in the shorter construct and two additional sites can be found in the −2.26 kb construct, when the sequence is analyzed using the SBE consensus sequence CAGAC, as postulated by Qin *et al.* [45].

The fact that the constructs comprising proximal promoter regions were not responsive to 1α,25(OH)_2_D_3_ is in line with recent findings on other 1α,25(OH)_2_D_3_ target genes, for which it was shown that results from assays using proximal promoter constructs were only modest or difficult to interpret. It has been put forward that multiple regulatory sequences, often located many kilobases from the TSS, are needed for expression control [46]. Recent findings on Smad action have established that Smad action depends on cooperation with “master transcription factors” [47], which are as yet undefined in monocytes and may be located at other regions of the CD69 gene or its 5′ flanking sequence.

Both, the combination of TGF-β/1α,25(OH)_2_D_3_ and PMA, are described to induce differentiation events in cells of the monocytic lineage [48], [49]. As constitutive expression of CD69 in CD14 positive monocytes is described [3], it is possible that induction of CD69 is a differentiational event. This is in line with the different basal CD69 mRNA levels in Mono Mac 6 and THP-1 together with the fact that THP-1 cells in contrast to Mono Mac 6, are negative for the monocytic marker CD14 [50], [51]. However, the mechanisms that lead to CD69 mRNA induction by TGF-β/1α,25(OH)_2_D_3_ and PMA seem to be diverse and synergism is strongly dependent on the time point that is considered. In conclusion, we show here that CD69 (mRNA) expression is cell-specifically regulated by TGF-β/1α,25(OH)_2_D_3_ in monocytic cells. This feature is in common with the gene encoding 5-lipoxygenase, the key enzyme in leukotriene formation. The parallelism supports the idea of a functional relation between the two proteins, which needs to be examined in further studies. Moreover, our results demonstrate that the combination of the physiological mediators TGF-β and 1α,25(OH)_2_D_3_ act on different genes by individual mechanisms and kinetics.

## References

[1] Sancho D, Gomez M, Sanchez-Madrid F (2005) CD69 is an immunoregulatory molecule induced following activation. Trends in Immunology 26: 136–140. 15745855 10.1016/j.it.2004.12.006

[2] Shiow LR, Rosen DB, Brdickova N, Xu Y, An J, et al (2006) CD69 acts downstream of interferon-alpha/beta to inhibit S1P1 and lymphocyte egress from lymphoid organs. Nature 440: 540–544. 16525420 10.1038/nature04606

[3] De Maria R, Cifone MG, Trotta R, Rippo MR, Festuccia C, et al (1994) Triggering of human monocyte activation through CD69, a member of the natural killer cell gene complex family of signal transducing receptors. J Exp Med 180: 1999–2004. 7964477 10.1084/jem.180.5.1999 PMC2191715

[4] Santos-Alvarez J, Goberna R, Sanchez-Margalet V (1999) Human leptin stimulates proliferation and activation of human circulating monocytes. Cell Immunol 194: 6–11. 10357875 10.1006/cimm.1999.1490

[5] Haeggstrom JZ, Funk CD (2011) Lipoxygenase and leukotriene pathways: biochemistry, biology, and roles in disease. Chem Rev 111: 5866–5898. 21936577 10.1021/cr200246d

[6] Ramirez R, Carracedo J, Castedo M, Zamzami N, Kroemer G (1996) CD69-induced monocyte apoptosis involves multiple nonredundant signaling pathways. Cell Immunol 172: 192–199. 8964080 10.1006/cimm.1996.0232

[7] Steinhilber D, Radmark O, Samuelsson B (1993) Transforming growth factor beta upregulates 5-lipoxygenase activity during myeloid cell maturation. Proc Natl Acad Sci U S A 90: 5984–5988. 8327471 10.1073/pnas.90.13.5984 PMC46851

[8] Brungs M, Radmark O, Samuelsson B, Steinhilber D (1995) Sequential induction of 5-lipoxygenase gene expression and activity in Mono Mac 6 cells by transforming growth factor beta and 1,25-dihydroxyvitamin D3. Proc Natl Acad Sci U S A 92: 107–111. 7816797 10.1073/pnas.92.1.107 PMC42826

[9] Heberden C, Denis I, Pointillart A, Mercier T (1998) TGF-beta and calcitriol. Gen Pharmacol 30: 145–151. 9502167 10.1016/s0306-3623(97)00271-1

[10] Carlberg C, Seuter S (2007) The vitamin D receptor. Dermatol Clin 25: 515–523, viii. 10.1016/j.det.2007.06.004 17903610

[11] Derynck R, Zhang YE (2003) Smad-dependent and Smad-independent pathways in TGF-beta family signalling. Nature 425: 577–584. 14534577 10.1038/nature02006

[12] Mu Y, Gudey SK, Landstrom M (2011) Non-Smad signaling pathways. Cell Tissue Res. 10.1007/s00441-011-1201-y 21701805

[13] Yamashita M, Fatyol K, Jin C, Wang X, Liu Z, et al (2008) TRAF6 mediates Smad-independent activation of JNK and p38 by TGF-beta. Mol Cell 31: 918–924. 18922473 10.1016/j.molcel.2008.09.002 PMC2621323

[14] Provost P, Doucet J, Hammarberg T, Gerisch G, Samuelsson B, et al (2001) 5-Lipoxygenase interacts with coactosin-like protein. J Biol Chem 276: 16520–16527. 11297527 10.1074/jbc.M011205200

[15] Wrana JL, Attisano L, Carcamo J, Zentella A, Doody J, et al (1992) TGF beta signals through a heteromeric protein kinase receptor complex. Cell 71: 1003–1014. 1333888 10.1016/0092-8674(92)90395-s

[16] Dennler S, Itoh S, Vivien D, ten Dijke P, Huet S, et al (1998) Direct binding of Smad3 and Smad4 to critical TGF beta-inducible elements in the promoter of human plasminogen activator inhibitor-type 1 gene. EMBO J 17: 3091–3100. 9606191 10.1093/emboj/17.11.3091 PMC1170648

[17] Herdick M, Steinmeyer A, Carlberg C (2000) Carboxylic ester antagonists of 1alpha,25-dihydroxyvitamin D(3) show cell-specific actions. Chem Biol 7: 885–894. 11094341 10.1016/s1074-5521(00)00036-3

[18] Bury Y, Ruf D, Hansen CM, Kissmeyer AM, Binderup L, et al (2001) Molecular evaluation of vitamin D3 receptor agonists designed for topical treatment of skin diseases. J Invest Dermatol 116: 785–792. 11348471 10.1046/j.1523-1747.2001.01332.x

[19] Yingling JM, Datto MB, Wong C, Frederick JP, Liberati NT, et al (1997) Tumor suppressor Smad4 is a transforming growth factor beta-inducible DNA binding protein. Mol Cell Biol 17: 7019–7028. 9372933 10.1128/mcb.17.12.7019 PMC232558

[20] Klan N, Steinhilber D (2003) Transient transfection of the human myeloid cell line Mono Mac 6 using electroporation. Biotechniques 34: 142–147. 12545551 10.2144/03341rr05

[21] Zufferey R, Nagy D, Mandel RJ, Naldini L, Trono D (1997) Multiply attenuated lentiviral vector achieves efficient gene delivery in vivo. Nat Biotechnol 15: 871–875. 9306402 10.1038/nbt0997-871

[22] Werz O, Brungs M, Steinhilber D (1996) Purification of transforming growth factor beta 1 from human platelets. Pharmazie 51: 893–896. 8985979

[23] Lopez-Cabrera M, Munoz E, Blazquez MV, Ursa MA, Santis AG, et al (1995) Transcriptional regulation of the gene encoding the human C-type lectin leukocyte receptor AIM/CD69 and functional characterization of its tumor necrosis factor-alpha-responsive elements. Journal of Biological Chemistry 270: 21545–21551. 7665567 10.1074/jbc.270.37.21545

[24] Santis AG, Lopez-Cabrera M, Sanchez-Madrid F, Proudfoot N (1995) Expression of the early lymphocyte activation antigen CD69, a C-type lectin, is regulated by mRNA degradation associated with AU-rich sequence motifs. European Journal of Immunology 25: 2142–2146. 7664776 10.1002/eji.1830250804

[25] Kanies CL, Smith JJ, Kis C, Schmidt C, Levy S, et al (2008) Oncogenic Ras and transforming growth factor-beta synergistically regulate AU-rich element-containing mRNAs during epithelial to mesenchymal transition. Mol Cancer Res 6: 1124–1136. 18644977 10.1158/1541-7786.MCR-07-2095 PMC2572152

[26] Castellanos MC, Munoz C, Montoya MC, Lara-Pezzi E, Lopez-Cabrera M, et al (1997) Expression of the leukocyte early activation antigen CD69 is regulated by the transcription factor AP-1. Journal of Immunology 159: 5463–5473. 9580241

[27] Ross S, Hill CS (2008) How the Smads regulate transcription. Int J Biochem Cell Biol 40: 383–408. 18061509 10.1016/j.biocel.2007.09.006

[28] Sorrentino A, Thakur N, Grimsby S, Marcusson A, von Bulow V, et al (2008) The type I TGF-beta receptor engages TRAF6 to activate TAK1 in a receptor kinase-independent manner. Nat Cell Biol 10: 1199–1207. 18758450 10.1038/ncb1780

[29] Hara T, Jung LK, Bjorndahl JM, Fu SM (1986) Human T cell activation. III. Rapid induction of a phosphorylated 28 kD/32 kD disulfide-linked early activation antigen (EA 1) by 12-o-tetradecanoyl phorbol-13-acetate, mitogens, and antigens. J Exp Med 164: 1988–2005. 2946796 10.1084/jem.164.6.1988 PMC2188483

[30] Cebrian M, Redondo JM, Lopez-Rivas A, Rodriguez-Tarduchy G, De Landazuri MO, et al (1989) Expression and function of AIM, an activation inducer molecule of human lymphocytes, is dependent on the activation of protein kinase C. Eur J Immunol. 19: 809–815. 10.1002/eji.1830190505 2786811

[31] Castellanos Mdel C, Lopez-Giral S, Lopez-Cabrera M, de Landazuri MO (2002) Multiple cis-acting elements regulate the expression of the early T cell activation antigen CD69. European Journal of Immunology 32: 3108–3117. 12385031 10.1002/1521-4141(200211)32:11<3108::AID-IMMU3108>3.0.CO;2-D

[32] Zhang DE, Hetherington CJ, Gonzalez DA, Chen HM, Tenen DG (1994) Regulation of CD14 expression during monocytic differentiation induced with 1 alpha,25-dihydroxyvitamin D3. J Immunol 153: 3276–3284. 7522257

[33] Pan Z, Hetherington CJ, Zhang DE (1999) CCAAT/enhancer-binding protein activates the CD14 promoter and mediates transforming growth factor beta signaling in monocyte development. J Biol Chem 274: 23242–23248. 10438498 10.1074/jbc.274.33.23242

[34] Jakobsson PJ, Steinhilber D, Odlander B, Radmark O, Claesson HE, et al (1992) On the expression and regulation of 5-lipoxygenase in human lymphocytes. Proc Natl Acad Sci U S A 89: 3521–3525. 1314391 10.1073/pnas.89.8.3521 PMC48900

[35] Karimi K, Gemmill TR, Lennartz MR (1999) Protein kinase C and a calcium-independent phospholipase are required for IgG-mediated phagocytosis by Mono-Mac-6 cells. J Leukoc Biol 65: 854–862. 10380910 10.1002/jlb.65.6.854

[36] Newton R, Stevens DA, Hart LA, Lindsay M, Adcock IM, et al (1997) Superinduction of COX-2 mRNA by cycloheximide and interleukin-1beta involves increased transcription and correlates with increased NF-kappaB and JNK activation. FEBS Lett 418: 135–138. 9414112 10.1016/s0014-5793(97)01362-8

[37] Diaz A, Chepenik KP, Korn JH, Reginato AM, Jimenez SA (1998) Differential regulation of cyclooxygenases 1 and 2 by interleukin-1 beta, tumor necrosis factor-alpha, and transforming growth factor-beta 1 in human lung fibroblasts. Exp Cell Res 241: 222–229. 9633531 10.1006/excr.1998.4050

[38] Yim D, Sotiriadis J, Kim KS, Shin SC, Jie HB, et al (2002) Molecular cloning, expression pattern and chromosomal mapping of pig CD69. Immunogenetics 54: 276–281. 12136339 10.1007/s00251-002-0464-6

[39] Seuter S, Sorg BL, Steinhilber D (2006) The coding sequence mediates induction of 5-lipoxygenase expression by Smads3/4. Biochem Biophys Res Commun 348: 1403–1410. 16919603 10.1016/j.bbrc.2006.08.011

[40] Yue J, Mulder KM (2000) Requirement of Ras/MAPK pathway activation by transforming growth factor beta for transforming growth factor beta 1 production in a Smad-dependent pathway. J Biol Chem 275: 30765–30773. 10843986 10.1074/jbc.M000039200

[41] Canonne-Hergaux F, Aunis D, Schaeffer E (1995) Interactions of the transcription factor AP-1 with the long terminal repeat of different human immunodeficiency virus type 1 strains in Jurkat, glial, and neuronal cells. J Virol 69: 6634–6642. 7474072 10.1128/jvi.69.11.6634-6642.1995 PMC189572

[42] Sorg BL, Klan N, Seuter S, Dishart D, Radmark O, et al (2006) Analysis of the 5-lipoxygenase promoter and characterization of a vitamin D receptor binding site. Biochim Biophys Acta 1761: 686–697. 16750418 10.1016/j.bbalip.2006.04.005

[43] Deng T, Karin M (1994) c-Fos transcriptional activity stimulated by H-Ras-activated protein kinase distinct from JNK and ERK. Nature 371: 171–175. 8072547 10.1038/371171a0

[44] Sandelin A, Wasserman WW (2005) Prediction of nuclear hormone receptor response elements. Mol Endocrinol 19: 595–606. 15563547 10.1210/me.2004-0101

[45] Qin H, Chan MW, Liyanarachchi S, Balch C, Potter D, et al (2009) An integrative ChIP-chip and gene expression profiling to model SMAD regulatory modules. BMC Syst Biol 3: 73. 19615063 10.1186/1752-0509-3-73 PMC2724489

[46] Pike JW, Meyer MB (2010) The vitamin D receptor: new paradigms for the regulation of gene expression by 1,25-dihydroxyvitamin D(3). Endocrinol Metab Clin North Am 39: 255–269, table of contents. 10.1016/j.ecl.2010.02.007 PMC2879406 20511050

[47] Mullen AC, Orlando DA, Newman JJ, Loven J, Kumar RM, et al (2011) Master transcription factors determine cell-type-specific responses to TGF-beta signaling. Cell 147: 565–576. 22036565 10.1016/j.cell.2011.08.050 PMC3212730

[48] Testa U, Masciulli R, Tritarelli E, Pustorino R, Mariani G, et al (1993) Transforming growth factor-beta potentiates vitamin D3-induced terminal monocytic differentiation of human leukemic cell lines. J Immunol 150: 2418–2430. 8383719

[49] Brungs M, Radmark O, Samuelsson B, Steinhilber D (1994) On the induction of 5-lipoxygenase expression and activity in HL-60 cells: effects of vitamin D3, retinoic acid, DMSO and TGF beta. Biochem Biophys Res Commun 205: 1572–1580. 7811238 10.1006/bbrc.1994.2846

[50] Ziegler-Heitbrock HW, Thiel E, Futterer A, Herzog V, Wirtz A, et al (1988) Establishment of a human cell line (Mono Mac 6) with characteristics of mature monocytes. Int J Cancer 41: 456–461. 3162233 10.1002/ijc.2910410324

[51] Morikawa M, Harada N, Soma G, Yoshida T (1990) Transforming growth factor-beta 1 modulates the effect of 1 alpha, 25-dihydroxyvitamin D3 on leukemic cells. In Vitro Cell Dev Biol 26: 682–690. 2384446 10.1007/BF02624424

