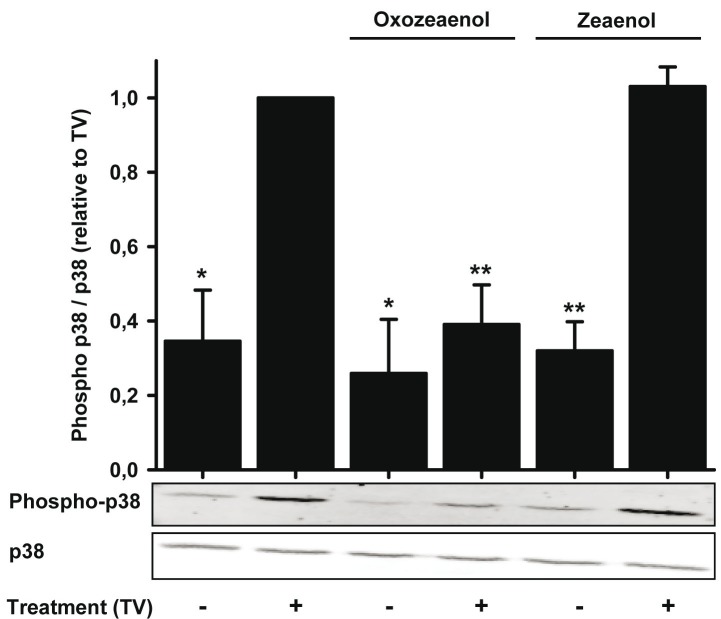# Correction: CD69 Is a TGF-β/1α,25-dihydroxyvitamin D_3_ Target Gene in Monocytes

**DOI:** 10.1371/annotation/b0f3fd01-7aad-498d-a104-d64d1f8deb29

**Published:** 2014-01-31

**Authors:** Thea K. Wöbke, Andreas von Knethen, Dieter Steinhilber, Bernd L. Sorg

In Figure 9B, the upper panel showing the phospho-p38 signals, is turned upside down. The correct Figure 9B can be found at the following link: 

**Figure:**